# Genomic prediction models for traits differing in heritability for soybean, rice, and maize

**DOI:** 10.1186/s12870-022-03479-y

**Published:** 2022-02-26

**Authors:** Avjinder S. Kaler, Larry C. Purcell, Timothy Beissinger, Jason D. Gillman

**Affiliations:** 1 Department of Crop, Soil, and Environmental Sciences, University of Arkansas, Fayetteville, AR 72704 USA; 2 Department of Crop Science & Center for Integrated Breeding Research, University of Goettingen, 37075 Goettingen, Germany; 3 Plant Genetics Research Unit, USDA-ARS, 205 Curtis Hall, University of Missouri, Columbia, MO 65211 USA

**Keywords:** Maize (*Zea mays* L.), Soybean (*Glycine max* L.), Rice (*Oryza sativa* L.), Genomic selection/prediction, Bayes B, Genomic estimated breeding values

## Abstract

**Background:**

Genomic selection is a powerful tool in plant breeding. By building a prediction model using a training set with markers and phenotypes, genomic estimated breeding values (GEBVs) can be used as predictions of breeding values in a target set with only genotype data. There is, however, limited information on how prediction accuracy of genomic prediction can be optimized. The objective of this study was to evaluate the performance of 11 genomic prediction models across species in terms of prediction accuracy for two traits with different heritabilities using several subsets of markers and training population proportions. Species studied were maize (*Zea mays*, L.), soybean (*Glycine max*, L.), and rice (*Oryza sativa*, L.), which vary in linkage disequilibrium (LD) decay rates and have contrasting genetic architectures.

**Results:**

Correlations between observed and predicted GEBVs were determined via cross validation for three training-to-testing proportions (90:10, 70:30, and 50:50). Maize, which has the shortest extent of LD, showed the highest prediction accuracy. Amongst all the models tested, Bayes B performed better than or equal to all other models for each trait in all the three crops. Traits with higher broad-sense and narrow-sense heritabilities were associated with higher prediction accuracy. When subsets of markers were selected based on LD, the accuracy was similar to that observed from the complete set of markers. However, prediction accuracies were significantly improved when using a subset of total markers that were significant at *P* ≤ 0.05 or *P* ≤ 0.10. As expected, exclusion of QTL-associated markers in the model reduced prediction accuracy. Prediction accuracy varied among different training population proportions.

**Conclusions:**

We conclude that prediction accuracy for genomic selection can be improved by using the Bayes B model with a subset of significant markers and by selecting the training population based on narrow sense heritability.

**Supplementary Information:**

The online version contains supplementary material available at 10.1186/s12870-022-03479-y.

## Background

Plant breeders observe the phenotypes of crops to choose desirable offspring in an aim to genetically improve target traits. Selection during the breeding process is a crucial step in crop breeding, with historical and current conventional plant breeding depending on phenotypic selection. The Selection-index (SI) method [1] selects multiple traits simultaneously based on a total score, is more efficient than selection for one trait at a time, and can improve aggregate genetic gain over time [2]. With advancements in computation, Henderson [3] proposed the best linear unbiased prediction (BLUP), which has become the most widely used method for genetic evaluation. High throughput genotyping methods have resulted in a large number of molecular markers that are available to assist in crop breeding. Although marker-assisted selection (MAS) is a popular method in molecular crop breeding [4], utilization of MAS has been limited in breeding programs because many of the important agronomic traits in crop breeding are complex and controlled by a large number of genes with small effects [5], making effective MAS difficult or impossible.

Genomic selection provides a potential advantage in crop breeding by accelerating the genetic improvement of crops per unit time through the reduction in cost per breeding cycle and shortening of the generation interval [6]. Additionally, genomic prediction can save labor costs compared to conventional breeding [7]. Meuwissen et al. [8] proposed a genomic selection method that uses genome-wide markers to estimate the effects of all loci and from which a genomic estimated breeding value (GEBV) can be computed to make the prediction for progeny of the target set with only genotypic data. A basic requirement for genomic prediction is that markers are distributed throughout the genome so that at least one marker is in linkage disequilibrium (LD) with each QTL [9]. All markers are used simultaneously to estimate effects using a “training” population [10]. Based on the training population, genomic prediction can predict GEBVs of individuals for selection. The GEBV of each individual can be estimated using markers whose effects can be estimated using a linear mixed model of the form y = Xβ + Zα + e, where y is a vector of standardized phenotypes, β is a vector of fixed effects, α is a vector of random effects for each marker, e is the random error, and X and Z are incidence matrices. When the number of predictors (markers) is much higher than the number of genotypes, fixed regression methods using ordinary least squares cannot be used for developing prediction models because of overfitting among predictors [11].

Numerous genomic prediction models have been developed for predicting phenotypes using large sets of genetic markers (relevant examples are listed in Additional File 1, Table S1). Variation resulting from hundreds or thousands of markers can be controlled by various shrinkage and variable selection methods. Models differ in various assumptions including the distribution of marker effects and marker variances. Methods such as ridge regression assume that marker effects are homogeneously distributed across the genome [12], whereas Bayesian methods allow for heterogeneity among markers, with some markers having effects coming from a different underlying distribution than others [13]. In methods such as BayesB, a prior probability distribution is used to select a proportion of markers with non-zero effects; in least absolute shrinkage and selection operator (LASSO) models, penalties are used to select markers with major effects [14]. BayesB and Bayesian LASSO methods can identify a subset of markers with large effects (variable selection) and use them for making predictions.

The utility of genomic prediction is expressed as the correlation between predicted and phenotypic values so that prediction of the individuals can be made accurately in earlier generations, with the aim to shorten selection cycles. Generally, it is assumed that the accuracy of prediction can be increased as more individuals are included in the training population and more markers are used in the prediction model. However, in practice this is not true in every case; increased size of the training population and increased number of markers in the prediction model may not lead to improved prediction accuracy (PA) [15] because of overfitting or due to the presence of non-informative individuals in a larger training population. Therefore, the first step in genomic prediction is to determine the size of the training population and the number of markers used in the appropriate prediction model to estimate the GEBVs with an aim to obtain high predictive ability. The second step is the validation and testing of the models to predict the phenotype of those lines that were not present in the training models [16]. The accuracy of a model for prediction is typically evaluated using cross-validation techniques under the assumption that random partitioning of the data results in independent training and testing sets [12, 17]. Both the manner in which training–testing partitions are constructed, as well as the level of relatedness among individuals, have effects on cross validation results [13].

Several factors affect the accuracy of genomic prediction including the size and genetic diversity of the training population, trait heritability, marker density, gene or marker effects, and the extent and distribution of LD between markers and QTL. Therefore, there is an ongoing need to understand how accuracy of different models reacts among crop species that vary in the LD decay as well as how prediction accuracy is affected by marker number, training population proportion, and trait heritability. The main objective of this study was to compare the accuracy of numerous genomic prediction models for several traits that differ in heritability for three crop species with different LD decays rates and contrasting genetic architecture, as well as testing the effect of several methods of sub-setting total marker number and the interaction with training population size.

## Results

### Descriptive statistics of phenotypes

Broad sense heritability and descriptive statistics of all traits for soybean [18, 19] maize [20], and rice [21] are presented in Table 1. There was a wide range of phenotypic variation for each trait. In soybean, CW ranged by 38.1% and δ^13^C ranged by 1.46%. In maize, the DT ranged by 30.5 days and the EH ranged by 128 cm. In rice, the PPP ranged by 1.89 panicles and the SPP ranged by 2.19 seeds (Table 1).

**Table 1:** Descriptive statistics and broad sense heritability of canopy wilting (CW) and carbon isotope ratio (*δ* ^13^C) in soybean, panicles per plant (PPP) and seeds per plant (SPP) in rice, and days to tasseling (DT) and ear height (EH) in maize

	Soybean^**a**^		Rice^**b**^		Maize^**c**^	
	CW (%)	***δ***^**13**^C (‰)	PPP	SPP	DT (days)	EH (cm)
**Mean**	16.99	−29.06	3.24	4.86	67.58	61.38
**Standard Deviation**	6.46	0.27	0.41	0.34	5.75	20.27
**Minimum**	7.5	−29.81	2.23	3.44	54.5	8
**Maximum**	45.63	−28.37	4.12	5.63	85	136
**Range**	38.13	1.46	1.89	2.19	30.5	128
**Count**	346	346	352	352	279	279
**Heritability (%)**	80	60	80	55	85	65

^a^CW data from Kaler et al. [18], and *δ* ^13^C from Kaler et al. [19]

^b^Data from Zhao et al. [21]

^c^Data from Wallace et al. [20]

### Markers distribution in subsets and narrow sense heritability

Tables S2, S3, and S4 (Additional File 1) show the distribution of markers across all chromosomes in different subsets of markers for all traits for all three crops. The number of markers was highest in maize compared to soybean and rice. To map a given trait with similar accuracy, maize requires a larger number of markers than soybean or rice because of faster LD decay. Both methods that were used to select subsets of markers reduced the marker number. Using subsets of markers based upon haplotype blocks decreased the number of markers by up to 58% (soybean), 26% (maize), and 37% (rice) (Table 2). Using subsets of markers based upon the probability (*P* = 0.05) of the association between a marker and given trait by FarmCPU [22] decreased the number of markers up to 97% (soybean), 96% (maize), and 98% (rice) (Table 2).

**Table 2:** Marker distribution in the different subsets of markers were selected based on the two methods: (1) when linkage disequilibrium between markers was correlated at *r* ≥ 0.90 (LD_90), *r* = 0.80 (LD_80), *r* = 0.70 (LD_70), *r* = 0.60 (LD_50) and (2) when SNP markers were significant with the respective traits at *P*-values of 50% (SNP_5), 10% (SNP_1), 5% (SNP_05), or non-significant (SNP_NS). The traits evaluated included canopy wilting (CW) and carbon isotope ratio (*δ* ^13^C) for soybean, panicles per plant (PPP) and seeds per plant (SPP) for rice, and days to tasseling ((DT) and ear height (EH) for maize

Crop	Trait	Complete	LD_90	LD_80	LD_70	LD_60	LD_50	SNP_5	SNP_1	SNP_05	SNP_NS
**Soybean**	**CW**	31,260	18,971	17,650	15,944	14,458	13,045	16,819	4106	2111	29,138
	***δ***^**13**^**C**	31,260	18,971	17,650	15,944	14,458	13,045	14,238	2174	919	30,332
**Rice**	**PPP**	34,848	28,390	26,808	25,437	23,910	22,107	15,983	2337	1043	33,804
	**SPP**	34,848	28,390	26,808	25,437	23,910	22,107	13,530	1554	674	34,169
**Maize**	**DT**	48,833	42,605	40,951	39,421	37,824	36,050	23,836	4277	2070	46,763
	**EH**	48,833	42,605	40,951	39,421	37,824	36,050	23,813	4257	2121	46,707

Marker based narrow sense heritability was estimated using marker information for all traits in the three crops for all different sets of markers and training population proportions. There was variation in *h*^*2*^ for all traits when using different subsets of markers for different training population proportions (Table 3). In general, for soybean and rice, *h*^*2*^ tended to increase as the subsets of markers based on *P-*values decreased from *P* = 0.50 to 0.05 (Table 3). This response was less evident in maize as *h*^*2*^ was largely unaffected by *P-*value, trait, or training population proportion. There was no discernable impact on *h*^*2*^ of limiting the subset of markers based on LD for any crop at any training population proportion.

**Table 3:** Marker based narrow sense heritability (*h*^*2*^) for canopy wilting (CW) and carbon isotope ratio (*δ* ^13^C) in soybean, and seeds per plant (SPP) and panicles per plant (PPP) in rice, and days to tasseling (DT) and ear height (EH) in maize using 10 sets of markers in different training-to-testing proportions (TPS)

Traits	TPS (%)	Subsets of markers^†^									
		Com	SNP_5	SNP_1	SNP_05	SNP_NS	LD_90	LD_80	LD_70	LD_60	LD_50
Soybean											
CW	90	74	76	77	78	76	75	76	76	75	75
	70	59	71	71	75	71	64	68	69	65	65
	50	79	79	80	79	79	78	79	79	78	78
δ^13^C	90	27	36	38	41	36	27	28	28	28	27
	70	38	46	45	48	46	38	39	39	38	37
	50	36	43	44	41	43	36	37	37	36	35
Rice											
SPP	90	36	49	51	52	27	34	34	34	33	34
	70	25	33	38	38	21	24	24	25	24	24
	50	28	42	38	43	23	28	27	27	26	26
PPP	90	61	78	81	80	57	61	61	61	61	61
	70	70	88	88	87	66	71	71	71	71	72
	50	77	84	82	77	74	76	76	76	76	76
Maize											
DT	90	80	85	85	83	76	81	81	81	81	81
	70	63	69	70	68	60	63	63	64	64	64
	50	97	99	99	98	88	98	98	98	98	98
EH	90	60	74	79	74	52	61	62	62	62	62
	70	52	64	63	61	44	52	52	52	53	52
	50	70	77	79	71	59	69	70	69	69	69

^†^Subsets of markers included a complete set (Com), SNP markers significant at *P*-values of 0.50 (SNP_5), 0.10 (SNP_1), 0.05 (SNP_05) or based upon linkage disequilibrium when the correlation coefficient between markers in a LD block were ≥ 0.90 (LD_90), 0.80 (LD_80), 0.70 (LD_70), 0.60 (LD_60), or 0.50 (LD_50)

### Genomic prediction accuracy in soybean, rice, and maize

**Fig. 1:**
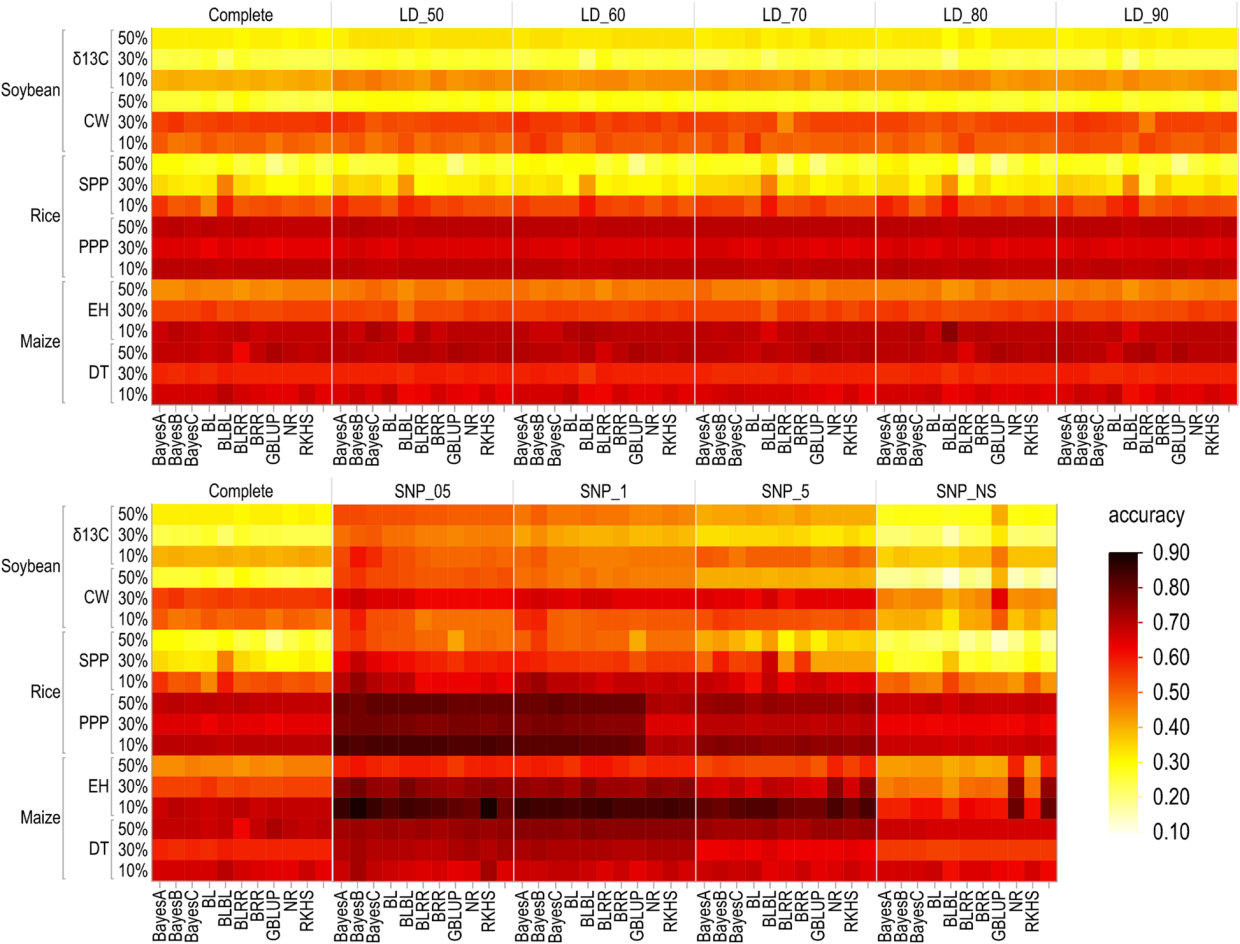
Prediction accuracies of 11 genomic prediction models in panicles per plant (PPP) and seeds per plant (SPP) in rice, days to tasseling (DT) and ear height (EH) in maize and canopy wilting (CW) and carbon isotope ratio (*δ* ^13^C) in soybean, using different subsets of markers, which were selected based on the two methods, linkage disequilibrium between markers and significant markers at different *P*-values using cross validation for three training-to-testing proportions (90:10%, 70:30%, and 50:50%)

Prediction accuracy of different genomic prediction models was compared for several traits differing in heritability in soybean, rice, and maize varying in LD decays rates using all markers, as well as several subsets of markers delimited by LD (five threshold subsets) or by marker significance (four threshold subsets) and training-to-testing population proportions. We found a difference in prediction accuracy for all traits in soybean, rice, and maize using different subsets of markers at three training-to-testing proportions (Fig. 1). Comparing different sets of markers, the subset SNP_05 yielded higher accuracy than the complete marker set, subsets selected based on the LD, and non-significant subset for all traits in soybean, rice, and maize.

**Fig. 2:**
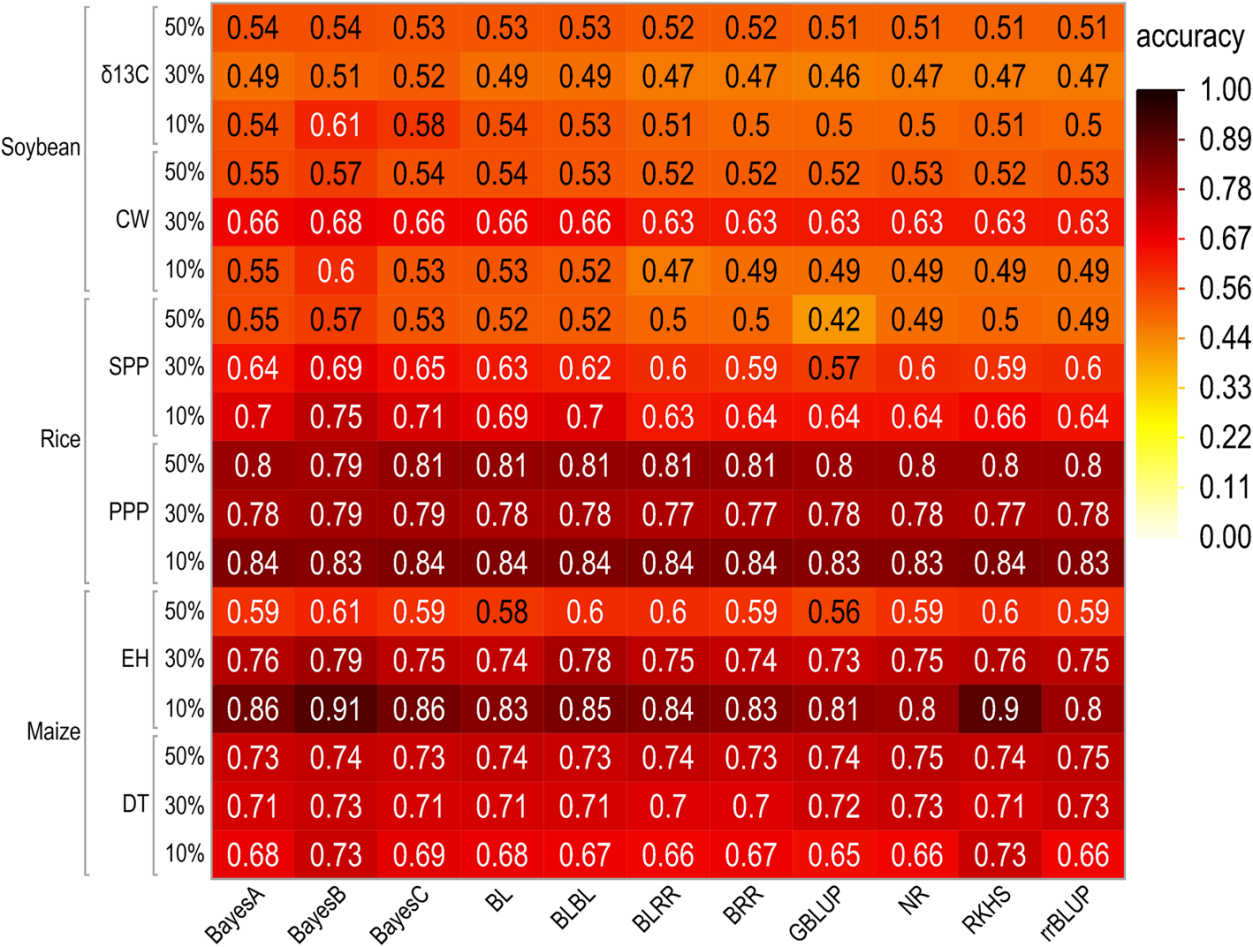
Prediction accuracies of 11 genomic prediction models in canopy wilting (CW) and carbon isotope ratio (*δ* ^13^C) in soybean, and panicles per plant (PPP) and seeds per plant (SPP) in rice, and days to tasseling (DT) and ear height (EH) in maize using a subset of significant markers at *P* < 0.05, using cross validation for three training-to-testing proportions (90:10%, 70:30%, and 50:50%)

Because the highest prediction accuracy was when using the SNP_05 subset, this subset was used to compare the different genomic prediction models (Fig. 2). For soybean, BayesB had the highest prediction accuracy for CW and *δ* ^13^C at all cross-validation levels. For SPP in rice, BayesB yielded the highest accuracy for all cross-validation levels. For PPP in rice, the accuracies of all models were similar for all of proportions between training and testing. For DT in maize, the highest prediction accuracy models were BayesB and RKHS at the 90:10 proportion. At the 70:30 and 50:50 proportions, the accuracies of all these models were mostly similar. For EH in maize, BayesB had the highest prediction accuracy at all training-to-testing proportions.

### Effect of the different training population proportions

Because a subset of significant markers selected at the significance level of *P* < 0.05 increased prediction accuracy in all selected traits of all crops, this subset was used to report the effect of the different training-to-testing population proportions (Fig. 2). Individuals were randomly assigned to training or testing sets, and this process was repeated 10 times. The effect of different testing population proportions on the prediction accuracy varied for different traits of these crops. For example, prediction accuracy of *δ* ^13^C and PPP was highest when the testing population proportion was 10% followed by 50 and 30% of the population. For CW, prediction accuracy was highest when the testing population proportion was 30% followed by 10 and 50% of the population. For SPP and EH, prediction accuracy was highest when the testing population proportion was 10% of the population followed by 30 and 50% of the population. For DT, prediction accuracy was highest when the testing population proportion was 30% followed by 50 and 10% of the population). These trends were similar for all the different sets of marker subsets (based upon significance threshold levels or LD decay rate; Additional File 1, Tables S6, S7, S8).

### Effect of marker density among species and traits on prediction accuracy

**Fig. 3:**
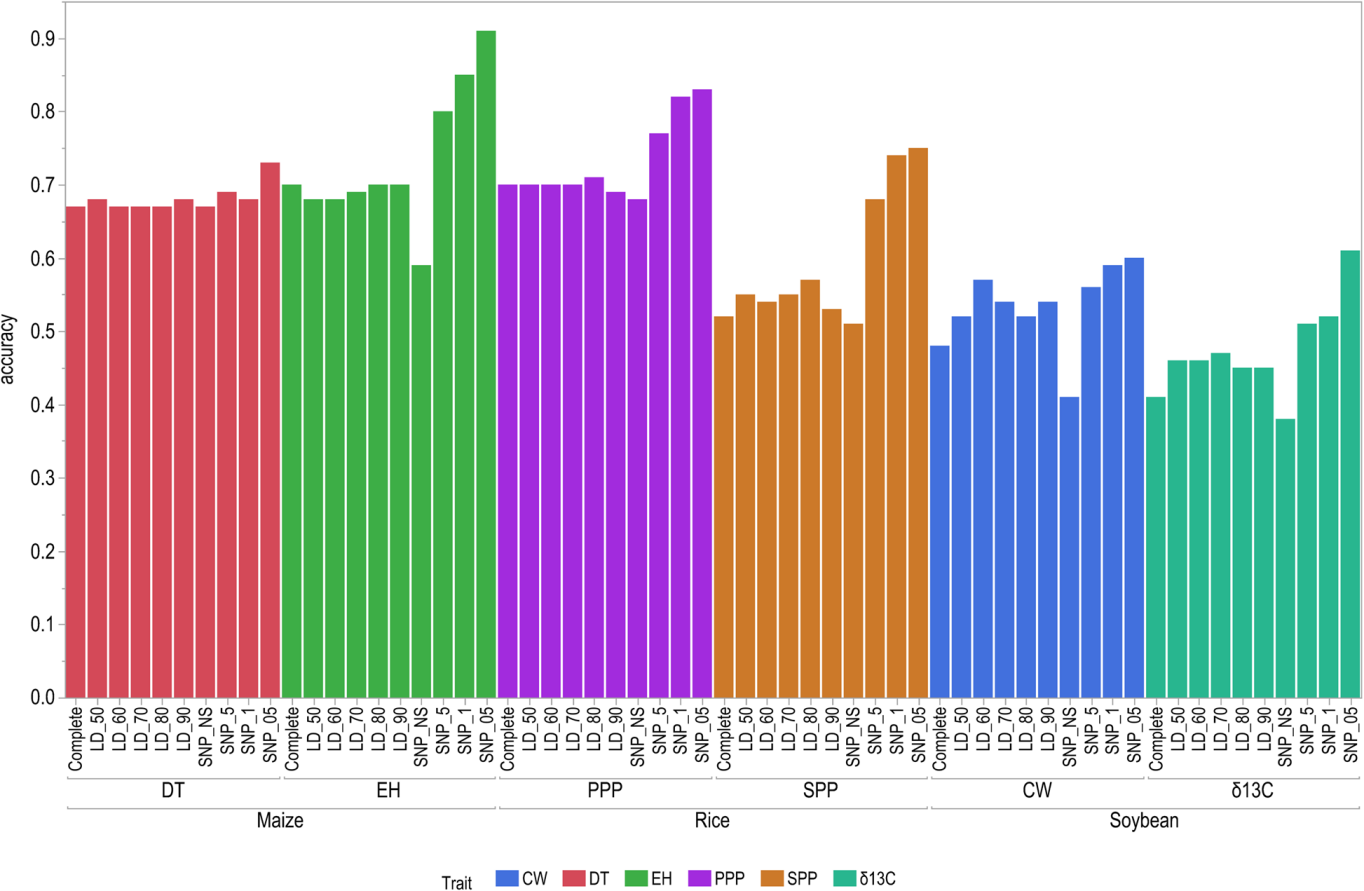
Effect of marker density of different subsets of markers, which were selected based on the two methods, linkage disequilibrium between markers and significant markers at different *P*-values, on the prediction accuracy of BayesB model for three traits including canopy wilting (CW) in soybean, seeds per plant (SPP) in rice, and ear height (EH) in maize using cross validation with a training-to-testing proportion of 90:10%

The effect of marker density is reported using BayesB model for each trait from each crop species including wilting (CW) in soybean, seeds per plant (SPP) in rice, and ear height (EH) in maize at the 90:10 training-to-testing proportion (Fig. 3). We used Bayes B model because it had a higher prediction accuracy than other models and had a lower computational requirement. There was no increase in accuracy among the non-significant subset of markers and marker subsets delimited by LD (five threshold subsets) among crop species compared with the full set of markers. When subsets of markers were selected based on significance, prediction accuracy was increased for all traits among species. Significant markers selected at the significance level of *P* < 0.1 and *P* < 0.05 had the highest prediction accuracy for all selected traits for all crops. Significant markers at *P* < 0.05 had the highest prediction accuracy for all traits except for DT at 50:50 proportion, where significant markers at *P* < 0.1 had the highest prediction accuracy.

### Effect of narrow sense heritability on prediction accuracy

We estimated *h*^*2*^ for all traits using all the different combinations of markers sets and three training-to-testing proportions (90:10, 70:30, and 50:50). (Table 3). We observed strong to moderate positive correlations between *h*^*2*^ and prediction accuracy of models under different sets of markers for all traits for all training population proportions (Additional File 1, Table S5). The effect of subsets of markers on *h*^*2*^ followed the same trend as the effect on the prediction accuracies.

## Discussion

This study evaluated the prediction accuracy of different genomic prediction models for crop species differing in LD and marker density, for traits differing in heritability, differences in marker density, and how proportions in the training-to-testing population affected prediction accuracy. To build a genomic prediction model, there is a need for a wide phenotypic variation [10], which was observed in this study. A basic assumption in genomic selection is that markers are distributed throughout the genome to provide sufficient coverage such that at least one marker is in LD with QTL that control the phenotypic variation. Genomic prediction models use all those effects to estimate GEBVs for progeny of the same or future generations [23].

We found that maize and rice had higher prediction accuracies than soybean. These crop species had different levels of LD/LD decay, which plays an important role in identifying marker-QTL associations [24]. Maize has a faster LD decay over physical distance compared to rice and soybean [18, 19, 25, 26]. Both maize and rice had more markers scattered over the genome than soybean, which increases the probability of having at least one marker in LD with a QTL [27]. The smaller number of markers with large effects in soybean may not explain all the genetic variance or may fail to capture small effect QTLs [28, 29]. Thavamanikumar et al. [30] reported similar results of difference in prediction accuracy among wheat populations varying in LD decay, which indicated that a high LD decay rate increases prediction accuracy.

In comparing the subsets of markers selected based on different LD levels and significance levels, we found there was a similar prediction accuracy for a complete set and subsets of markers selected based on LD. Thus, a haplotype block performed similar to a single marker. Adding more random markers in these conditions did not improve accuracy but may have increased error or noise. Poland et al. [31] and Spindel et al. [32] evaluated random subsets of markers and observed no change in accuracy. Prediction accuracies were increased when markers were selected that had some association with the phenotype instead of using all markers that may have created noise or error. In addition to subsets of markers evaluated at *P*-values of 0.05, 0.10, and 0.50, we also compared the effect of marker density of subsets of markers selected at 13 different levels of significance on prediction accuracy (Additional File 2, Fig. S1). Prediction accuracy increased as the significance level of markers decreased until *P* < 0.05, but there was no further increase in accuracy at lower *P* values. We conclude that significant markers should be selected up to a probability/significance level where they still have adequate genomic coverage. These results are consistent with previous research [30] in which there was greater accuracy when using QTL-linked markers than when using a random set of markers. The importance of including markers identified from QTL and association studies in prediction models was demonstrated when the QTL-linked markers were excluded from the models and there was a lower accuracy compared to other sets of markers [30].

As expected, higher prediction accuracies were observed for high heritability traits compared to low heritability traits. Similar results were observed in other studies where there was a strong relationship between prediction accuracy and trait heritability [27, 33–35]. Similar to broad-sense heritability, marker based narrow sense heritability varied among the traits in this study. There were strong positive correlations between marker based narrow sense heritability and prediction accuracy for all traits for all training-to-testing population proportions, indicating that marker based narrow sense heritability might be associated with prediction accuracy. Similar to accuracy, subsets of markers selected based on the different significance levels increased marker based narrow sense heritability, but LD based subsets of markers did not. Extra markers might be associated with an increase in error or noise from LD based subsets. We conclude that selecting training population proportions based on marker based narrow sense heritability may generally improve prediction accuracy.

Among all models, BayesB performed better than or equal to all other models, for all traits in all three crop species. The BayesB model performs both shrinkage and variable selection on markers included in the model [36]. In the BayesB model, the prior probability of a marker having a non-null effect (π) was set at 0.05, which might be associated with higher predictive ability values compared to higher prior setting. Results from this study agree with other studies, indicating that selecting a model that performs specific shrinkage and variable selection would improve prediction accuracy. For example, Bayesian Lasso and BayesB share some characteristics, and these models performed better than GBLUP, which assumes equal variance for each marker [23, 27]. The better performance of BayesB over other models was highly dependent on the presence of large QTL effects, which was demonstrated by Daetwyler et al. [14] through simulations. Several studies reported the better performance of BayesB over all models in genomic prediction of traits that are controlled by a few loci with large effects [14, 37, 38]. Habier et al. [24] provided another comparison between BayesB and rrBLUP models, indicating that BayesB uses LD between markers and QTL in making predictions, where RR-BLUP mainly captures the genetic relationships. Accuracies of the models using LD between markers and QTL persist for several generations, whereas accuracies of the models using genetic relationships decay over generations [24]. In this study, prediction accuracy was affected more by LD between the markers and QTLs than the genetic relationships.

The effect of different training population proportions on prediction accuracy was compared in this study for all traits in three crops by randomly repeating the simulations 10 times. We observed a difference in prediction accuracy among training population proportions that might be due to the random selection of the training population. A random population could have a different genetic diversity or population structure, which could have different marker-QTLs associations or marker effect sizes. Similar results of varied prediction accuracies due to different training populations were observed in other studies [39]. Charmet et al. [40] observed that predicted accuracy was not improved with an increase of the training population proportion. However, de Azevedo Peixoto et al. [41] observed an increase in prediction accuracy when the training population proportion increased.

## Conclusions

In this study, we compared the prediction accuracy of different genomic prediction models for several traits differing in heritability in three crops varying in LD decays rates with contrasting genetic architecture using several subsets of markers and training population proportions. Among all models, Bayes B performed better than or equally well as all other models for all traits in three crop species. Higher prediction accuracy was observed in maize with a faster LD decay compared to slower LD decay in soybean and rice. Traits with higher broad or narrow sense heritability had higher prediction accuracy. Instead of using a complete set of markers, selecting subsets of markers based on the significance level increased prediction accuracy. Prediction accuracy was greatest for all crops when using a subset of markers that were significant at *P* ≤ 0.05. In contrast, subsets of markers selected based on the LD level did not show any change in the accuracy. Different training population proportions varied prediction accuracy for all traits in three crops.

## Materials and methods

### Plant materials and phenotypic traits

Data sets for three plant species that vary in LD decay rates were selected for our experiments: soybean (*Glycine max* L.), maize (*Zea mays* L.), and rice (*Oryza sativa* L.). For each crop, two traits were used that varied in broad-sense heritability (H^2^). For soybean, these traits included canopy wilting (CW, H^2^ = 80%, [19]) and carbon isotope ratio (*δ* ^13^C, H^2^ = 60%, [18]). For maize, traits evaluated were days to tasseling (DT, H^2^ = 85%) and ear height (EH, H^2^ = 65%) [20]. Lastly for rice, we evaluated panicles per plant (PPP, H^2^ = 80%) and seeds per plant (SPP, H^2^ = 55%) [21].

Soybean data consisted of 346 accessions that were used for association mapping of CW [19] and *δ* ^13^C [18]. Rice data consisted of 352 accessions that were obtained from the rice diversity database [21]. Maize data consisted of 279 accessions that were obtained from the Panzea database website [20]. Both maize (https://www.panzea.org/data) and rice data (http://ricediversity.org/data/) were publicly available and soybean data are included herein (Additional file 3).

### Genotypic data

For all three crops, genotypic data were comprised of single nucleotide polymorphisms (SNPs). In soybean, SNP data were obtained using the application of Illumina Infinium SoySNP50K iSelect SNP BeadChip that provided 42,509 SNPs for all 346 accessions ([42, 43], and datasets supporting the conclusions of this article are included within the article (Additional Files 3 and 4).

Additional file 3). In maize, SNP data were obtained using the application of Illumina MaizeSNP50 BeadChip that provided 50,896 SNPs for all 273 accessions [20]. In rice, SNP data of 44,100 markers were obtained from two projects: *Oryza*SNP project, an oligomer array-based re-sequencing effort using Perlegen Sciences technology and BAC clone Sanger sequencing of wild species from OMAP project [21]. Quality control checks for the three species consisted of eliminating monomorphic markers, markers with minor allele frequency (MAF) ≤ 5*%*, and markers with a missing rate higher than 10*%*. Remaining marker datasets were imputed using an LD-kNNi method, which is based on a k-nearest-neighbor-genotype [44]. The final complete SNP marker datasets consisted of 31,260 SNPs for soybean, 48,833 SNPs for maize, and 34,848 SNPs for rice. A pairwise SNP LD decay among the markers for these crops was estimated, which indicated that the decay of LD to r^2^ = 0.25 level was much faster in maize (1 kb) than soybean (150 kb in euchromatic and 5 kb in heterochromatic regions) or rice (123 kb).

### Genomic prediction models

Eleven different statistical models were compared for genomic predictions. These models differ with respect to assumptions about the markers as described in Additional File 1, Table S1. Prediction models were tested using different packages including sommer, rrBLUP, BGLR, plyr, MCMCglmm, EMMREML, and BLR in the R program.

### Testing subsets of markers in prediction models

Ten different marker subsets were compared to evaluate the effects of marker distribution across the genome on the prediction accuracy. Subsetting markers was done based on two approaches: 1) linkage disequilibrium between markers and 2) markers that met a significance threshold. Linkage disequilibrium between markers, which defines a haplotype block, was evaluated using the correlation coefficient between alleles at a pair of genetic loci. Five LD based subsets of markers were selected from the haplotype block that was made using a correlation coefficient (r) of *r ≥* 0.90, 0.80 ≤ *r* < 0.90, 0.70 ≤ *r* < 0.80, 0.60 ≤ *r* < 0.70, and 0.50 ≤ *r* < 0.60 between alleles. For simplicity, these subsets are subsequently referred to as 0.90, 0.80, 0.70, 0.60, and 0.50, respectively. For example, if a haplotype block consisted of five SNPs that were linked with each other at *r ≥* 0.90, then only one SNP out of five was kept in the subset of markers.

In addition to selecting marker subsets based upon haplotype blocks, we also selected subsets of markers based on the *P*-value of the significant association of markers with a trait at probability levels of *P* < 0.5 (SNP_5), *P* < 0.1 (SNP_1), and *P* < 0.05 (SNP_05). The significant association between markers and traits was conducted using the Fixed and random model Circulating Probability Unification (FarmCPU) model [22]. One subset of markers consisted of non-significant SNP markers at *P* > 0.05 (SNP_NS). The F-test for two-samples of variance was conducted to compare the significant effect between the different sets of markers.

### Testing the size of the training population and model validation

Three different sizes of training populations among these species were evaluated to determine the effect of the training population proportion on the genomic prediction accuracy. These three training population proportions consisted of 90 training to 10 testing set, 70 training to 30 testing set, and 50 training to 50 testing set. Random assignments of individuals to training and testing sets were repeated 10 times and the average value of the prediction accuracy are reported in this work. The correlation coefficient (r) between the GEBVs and observed phenotypic values was used to determines predictive ability (r), which was used as the indicator of prediction accuracy in this paper. This approach has been used previously by several groups [45, 46].

### Estimation of narrow sense heritability

Marker-based narrow sense heritability (*h*^*2*^) was estimated to understand the variation and trend of predictive ability across traits [47] using the GAPIT R package. In the GAPIT package, the MLM model can be described as follows: *Y* = *Xβ* + *Zu* + *e*, where Y is the vector of observed phenotypes; β is an unknown vector containing fixed effects, including the genetic marker, population structure (Q), and the intercept; u is an unknown vector of random additive genetic effects from multiple background QTLs for individuals/lines; X and Z are the known design matrices; and e is the unobserved vector of residuals. The u and e vectors are assumed to be normally distributed with a null mean and a variance of: $$Var\ \left(\left.\begin{array}{c}u\\ {}e\end{array}\right)\right.=\left(\begin{array}{cc}G& 0\\ {}0& R\end{array}\right)$$, where G = σ^2^_a_K with σ^2^ a as the additive genetic variance and K as the kinship matrix. Homogeneous variance was assumed for the residual effect (i.e., R = σ^2^_e_I, where σ^2^_e_ is the residual variance). The proportion of the total variance explained by the genetic variance is defined as marker-based narrow sense heritability, which was calculated for all traits using different subsets of markers and different training-to-testing population proportions.

## Supplementary Information

**Additional file 1: Table S1.** Main features of thirteen genomic prediction models. **Table S2.** Markers distribution in the different subsets of markers, which were selected based on the two methods, linkage disequilibrium (LD) between markers and significant markers, in soybean for two traits including canopy wilting (CW) and carbon isotope ratio (*δ* ^13^C). **Table S3.** Markers distribution in the different subsets of markers, which were selected based on the two methods, linkage disequilibrium (LD) between markers and significant markers, in maize for two traits including days to tasseling (DT) and ear height (EH). **Table S4.** Markers distribution in the different subsets of markers, which were selected based on the two methods, linkage disequilibrium (LD) between markers and significant markers, in rice for two traits including panicles per plant (PPP) and seeds per plant (SPP). **Table S5.** Correlations of narrow sense heritabilities and prediction accuracies of 11 different genomic prediction models for canopy wilting (CW) carbon isotope ratio (*δ* ^13^C), seeds per plant (SPP), panicles per plant (PPP), and days to tasseling (DT) and ear height (EH) at different training-to-testing proportions (90/10%, 70/30%, & 50/50%). **Table S6.** Prediction accuracy of genomic models for canopy wilting (CW) and carbon isotope ratio (*δ* ^13^C) in soybean, and seeds per plant (SPP) and panicles per plant (PPP) in rice, and days to tasseling (DT) and ear height (EH) in maize using different subsets of markers at the 90/10% training-to-testing proportion. **Table S7.** Prediction accuracy of genomic models for canopy wilting (CW) and carbon isotope ratio (*δ* ^13^C) in soybean, and seeds per plant (SPP) and panicles per plant (PPP) in rice, and days to tasseling (DT) and ear height (EH) in maize using different subsets of markers at the 70/30% training-to-testing proportion. **Table S8.** Prediction accuracy of genomic models for canopy wilting (CW) and carbon isotope ratio (*δ* ^13^C) in soybean, and seeds per plant (SPP) and panicles per plant (PPP) in rice, and days to tasseling (DT) and ear height (EH) in maize using different subsets of markers at the 50/50% training-to-testing proportion.

**Additional file 2: Fig. S1.** Prediction accuracies for carbon isotope ratio (*δ13*) in soybean using BayesB model and a training-to-testing cross-validation proportion of 90:10%. Thirteen sets of marker subsets were selected based on the different significant levels including *P* < 0.001, *P* < 0.0025, *P* < 0.005, *P* < 0.0075, *P* < 0.01, *P* < 0.025, *P* < 0.05, *P* < 0.075, *P* < 0.1, *P* < 0.25, *P* < 0.5, *P* < 0.75, and complete set.

**Additional file 3. **Zip file containing genotypic and phenotypic data files for three datasets: soybean, two traits (*δ* ^13^C and CW) and 42,509 SNPs for 346 accessions; maize, two traits (DT and EH) and 50,896 SNPs for 273 accessions; rice, two traits (PPP and SPP) and 44,100 markers from two projects: *Oryza*SNP project and OMAP project.

**Additional file 4.** Zip file containing R scripts used in this study.

## Data Availability

All data analyzed during this study are included in this published article in are available in Additional file 3. Scripts used in our analyses are available in Additional file 4.

## Abbreviations

QTL: Quantitative trait locus/loci
LD: Linkage disequilibrium
SNP: Single nucleotide polymorphism
GEBV: Genomic estimated breeding value
BLUP: Best linear unbiased prediction
MAS: Marker-assisted selection
LASSO: Least absolute shrinkage and selection operator
PA: Prediction accuracy
CW: Canopy wilting
δ13C: Carbon isotope ratio (δ13C)
PPP: Panicles per plant
SPP: And seeds per plant
DT: Days to tasseling
EH: Ear height
