# Supplementary material for: Genomic prediction models for traits differing in heritability for soybean, rice, and maize
Source: BMC Plant Biol. 2022 Feb 26;22:87. doi: 10.1186/s12870-022-03479-y (PMC8881851; doi:10.1186/s12870-022-03479-y)
Supplement: Supplementary file 1 — Additional file 1: Table S1. Main features of thirteen genomic prediction models. Table S2. Markers distribution in the different subsets of markers, which were selected based on the two methods, linkage disequilibrium (LD) between markers and significant markers, in soybean for two traits including canopy wilting (CW) and carbon isotope ratio (δ 13C). Table S3. Markers distribution in the different subsets of markers, which were selected based on the two methods, linkage disequilibrium (LD) between markers and significant markers, in maize for two traits including days to tasseling (DT) and ear height (EH). Table S4. Markers distribution in the different subsets of markers, which were selected based on the two methods, linkage disequilibrium (LD) between markers and significant markers, in rice for two traits including panicles per plant (PPP) and seeds per plant (SPP). Table S5. Correlations of narrow sense heritabilities and prediction accuracies of 11 different genomic prediction models for canopy wilting (CW) carbon isotope ratio (δ 13C), seeds per plant (SPP), panicles per plant (PPP), and days to tasseling (DT) and ear height (EH) at different training-to-testing proportions (90/10%, 70/30%, & 50/50%). Table S6. Prediction accuracy of genomic models for canopy wilting (CW) and carbon isotope ratio (δ 13C) in soybean, and seeds per plant (SPP) and panicles per plant (PPP) in rice, and days to tasseling (DT) and ear height (EH) in maize using different subsets of markers at the 90/10% training-to-testing proportion. Table S7. Prediction accuracy of genomic models for canopy wilting (CW) and carbon isotope ratio (δ 13C) in soybean, and seeds per plant (SPP) and panicles per plant (PPP) in rice, and days to tasseling (DT) and ear height (EH) in maize using different subsets of markers at the 70/30% training-to-testing proportion. Table S8. Prediction accuracy of genomic models for canopy wilting (CW) and carbon isotope ratio (δ 13C) in soybean, and seeds per plan [file 12870_2022_3479_MOESM1_ESM.docx]

**Table S1.** Main features of thirteen genomic prediction models

| **Acronym*** | **Features** | **Ref** |
| --- | --- | --- |
| Bayes A | - Utilizes an inverse chi-square (x2) on marker variances yielding a scaled t-distribution for marker effects. - Similar to BL and in contrast to BRR, it shrinks tiny marker effects towards zero and larger values survive. | [1] |
| Bayes B | - Similar to Bayes A, uses an inverse x2 resulting in scaled t-distribution. - Unlike Bayes A, utilizes both shrinkage and variable selection. | [1] |
| Bayes C | - Applies both shrinkage and variable selection methods. - Characterized by a Gaussian distribution. - Bayes B and Bayes C consist of point of mass at zero in their slab priors. | [2] |
| BLBL_BLR | - Bayesian lasso uses the Laplace (double exponential, DE) distribution, where the prior assigned to marker effects and all marker effects are assumed to be independently and identically distributed. This prior assigns the same variance or prior uncertainty to all marker effects. This prior possesses thicker tails than the normal prior. Bayesian lasso removes markers from the model, contrary to what happens in variable selection approaches. Bayesian lasso is expected to shrink effects more strongly toward zero than the Gaussian prior, as opposed to inducing sparsity in the strict sense of the Lasso. | [3] |
| BLRR_BLR | - Induces homogeneous shrinkage of all marker effects towards zero and yields a Gaussian distribution of marker effects. - Similar to RR-BLUP, there is a problem of QTL linkages to the marker. | [2] |
| BL_BGLR | - Modified version of BLR, has same assumptions as BLBL_BLR. | [3] |
| BRR_BGLR | - Modified version of BLR, has same assumptions as BLRR_BLR. | [2] |
| NR | - Fit mixed models with the advantage of specifying the variance-covariance structure for the random effects and specify heterogeneous variances using more than one variance component and allowing specification of covariance structures. - Uses Direct-Inversion Newton-Raphson algorithm. | [4] |
| RKHS | - Based on genetic distance and a kernel function with a smoothing parameter to regulate the distribution of QTL effects. - Effective for detecting nonadditive gene effects. | [5] |
| rrBLUP | - Assumes markers have equal variances with small but non-zero effects.   Applies homogeneous shrinkage of predictors towards zero but allows for markers to have uneven effects.   - Computed from a realized-relation matrix based on markers. - Some QTL are in LD to marker loci, whereas others are not. | [1] |
| GBLUP | Assigns common variance to all loci and treats them as equal.  Uses a genomic relationship matrix instead of the conventional pedigree-derived numerator relationship matrix. | [6] |

*Bayes A, Bayes B, Bayes C, Bayesian LASSO (BL) using Bayesian Generalized Linear Regression R package (BGLR_R), and Bayesian Ridge Regression (BRR) using BGLR_R, Bayesian LASSO (BLBL) using Bayesian Linear Regression R package (BLR_R), and Bayesian Ridge Regression (BLRR) using BLR_R,  ridge regression–best linear unbiased prediction (rrBLUP),  Reproducing Kernel Hilbert Spaces (RKHS) regression, Mixed model using Newton-Raphson algorithm (NR), and Genomic best linear unbiased prediction (GBLUP).

References

1. Meuwissen TH, Hayes BJ, Goddard ME: **Prediction of total genetic value using genome-wide dense marker maps**. *Genetics* 2001, **157**(4):1819-1829.

2. de Los Campos G, Perez P, Vazquez AI, Crossa J: **Genome-enabled prediction using the BLR (Bayesian Linear Regression) R-package**. *Genome-Wide Association Studies and Genomic Prediction* 2013:299-320.

3. Pérez P, de Los Campos G: **Genome-wide regression and prediction with the BGLR statistical package**. *Genetics* 2014, **198**(2):483-495.

4. Covarrubias-Pazaran G: **Genome-Assisted Prediction of Quantitative Traits Using the R Package sommer**. *PLoS One* 2016, **11**(6):e0156744.

5. Gianola D, Van Kaam JB: **Reproducing kernel Hilbert spaces regression methods for genomic assisted prediction of quantitative traits**. *Genetics* 2008, **178**(4):2289-2303.

6. VanRaden PM: **Efficient methods to compute genomic predictions**. *J Dairy Sci* 2008, **91**(11):4414-4423.

**Table S2.** Markers distribution in the different subsets of markers, which were selected based on the two methods, linkage disequilibrium (LD) between markers and significant markers, in soybean for two traits including canopy wilting (CW) and carbon isotope ratio ($\delta$^13^C).

|  |  |  | **LD** |  |  |  |  |  |  | **CW** |  |  |  |  | $\boldsymbol{\delta}$**^13^C** |  |  |
| --- | --- | --- | --- | --- | --- | --- | --- | --- | --- | --- | --- | --- | --- | --- | --- | --- | --- |
| **CHR** | **Complete** | **LD_90** | **LD_80** | **LD_70** | **LD_60** | **LD_50** |  | **SNP_5** | **SNP_1** | **SNP_05** | **SNP_NS** |  | **SNP_5** | **SNP_1** | **SNP_05** | **SNP_NS** |  |
| 1 | 1238 | 729 | 617 | 539 | 476 | 434 |  | 694 | 146 | 64 | 1174 |  | 628 | 87 | 48 | 1190 |  |
| 2 | 1924 | 1230 | 1084 | 946 | 830 | 738 |  | 1016 | 239 | 103 | 1818 |  | 648 | 60 | 25 | 1894 |  |
| 3 | 1292 | 949 | 846 | 781 | 712 | 665 |  | 635 | 128 | 60 | 1232 |  | 609 | 82 | 24 | 1267 |  |
| 4 | 1415 | 950 | 874 | 816 | 733 | 670 |  | 668 | 130 | 40 | 1375 |  | 796 | 178 | 93 | 1322 |  |
| 5 | 1352 | 832 | 724 | 650 | 576 | 535 |  | 801 | 194 | 120 | 1232 |  | 554 | 89 | 29 | 1323 |  |
| 6 | 1360 | 39 | 845 | 763 | 702 | 632 |  | 879 | 205 | 117 | 1241 |  | 639 | 88 | 33 | 1327 |  |
| 7 | 1593 | 1032 | 895 | 835 | 762 | 682 |  | 862 | 229 | 116 | 1477 |  | 637 | 60 | 29 | 1564 |  |
| 8 | 1884 | 1013 | 855 | 752 | 693 | 614 |  | 776 | 177 | 106 | 1778 |  | 1036 | 217 | 94 | 1790 |  |
| 9 | 1448 | 956 | 858 | 796 | 722 | 671 |  | 665 | 125 | 57 | 1391 |  | 633 | 74 | 39 | 1409 |  |
| 10 | 1622 | 948 | 837 | 771 | 703 | 633 |  | 799 | 119 | 63 | 1559 |  | 622 | 149 | 90 | 1531 |  |
| 11 | 1220 | 726 | 651 | 597 | 552 | 483 |  | 690 | 160 | 92 | 1128 |  | 563 | 64 | 26 | 1194 |  |
| 12 | 1094 | 628 | 535 | 483 | 435 | 396 |  | 635 | 151 | 82 | 1012 |  | 461 | 42 | 20 | 1074 |  |
| 13 | 2019 | 1307 | 1189 | 1080 | 986 | 906 |  | 1160 | 343 | 151 | 1868 |  | 885 | 126 | 30 | 1989 |  |
| 14 | 1587 | 1013 | 918 | 782 | 697 | 621 |  | 793 | 193 | 121 | 1460 |  | 629 | 66 | 12 | 1575 |  |
| 15 | 1911 | 1183 | 1036 | 925 | 857 | 770 |  | 1126 | 305 | 149 | 1762 |  | 893 | 169 | 86 | 1825 |  |
| 16 | 1436 | 1093 | 1012 | 918 | 823 | 741 |  | 814 | 250 | 131 | 1305 |  | 733 | 154 | 71 | 1365 |  |
| 17 | 1586 | 1021 | 903 | 817 | 749 | 688 |  | 906 | 195 | 101 | 1485 |  | 890 | 239 | 84 | 1502 |  |
| 18 | 2595 | 1706 | 1548 | 1393 | 1285 | 1153 |  | 1581 | 481 | 245 | 2350 |  | 1310 | 127 | 48 | 2546 |  |
| 19 | 1624 | 855 | 728 | 646 | 584 | 499 |  | 833 | 233 | 143 | 1481 |  | 558 | 59 | 20 | 1604 |  |
| 20 | 1060 | 761 | 695 | 654 | 581 | 514 |  | 486 | 103 | 50 | 1010 |  | 514 | 44 | 18 | 1041 |  |
| **Total** | 31260 | 18971 | 17650 | 15944 | 14458 | 13045 |  | 16819 | 4106 | 2111 | 29138 |  | 14238 | 2174 | 919 | 30332 |  |
|  |  |  |  |  |  |  |  |  |  |  |  |  |  |  |  |  |  |

**Table S3.** Markers distribution in the different subsets of markers, which were selected based on the two methods, linkage disequilibrium (LD) between markers and significant markers, in maize for two traits including days to tasseling (DT) and ear height (EH).

|  |  |  | **LD** |  |  |  |  |  |  | **DT** |  |  |  | **EH** |  |  |
| --- | --- | --- | --- | --- | --- | --- | --- | --- | --- | --- | --- | --- | --- | --- | --- | --- |
| **CHR** | **Complete** | **LD_90** | **LD_80** | **LD_70** | **LD_60** | **LD_50** |  | **SNP_5** | **SNP_1** | **SNP_05** | **SNP_NS** |  | **SNP_5** | **SNP_1** | **SNP_05** | **SNP_NS** |
| 1 | 7592 | 6687 | 6448 | 6169 | 5936 | 5658 |  | 3817 | 702 | 341 | 7251 |  | 3801 | 715 | 349 | 7243 |
| 2 | 5732 | 5056 | 4869 | 4690 | 4505 | 4296 |  | 2541 | 365 | 180 | 5552 |  | 2685 | 494 | 245 | 5487 |
| 3 | 5493 | 4782 | 4582 | 4413 | 4217 | 4016 |  | 2727 | 481 | 217 | 5276 |  | 2642 | 454 | 227 | 5262 |
| 4 | 5373 | 4583 | 4385 | 4218 | 4045 | 3866 |  | 2564 | 410 | 207 | 5166 |  | 2623 | 431 | 202 | 5171 |
| 5 | 5326 | 4613 | 4409 | 4253 | 4072 | 3852 |  | 2655 | 503 | 238 | 5088 |  | 2604 | 482 | 248 | 5077 |
| 6 | 3988 | 3533 | 3403 | 3297 | 3155 | 3014 |  | 1858 | 340 | 165 | 3823 |  | 1950 | 364 | 189 | 3799 |
| 7 | 4056 | 3510 | 3373 | 3267 | 3144 | 3004 |  | 1918 | 369 | 192 | 3864 |  | 2007 | 318 | 161 | 3895 |
| 8 | 4163 | 3565 | 3425 | 3292 | 3177 | 3067 |  | 2040 | 305 | 138 | 4025 |  | 2018 | 348 | 153 | 4010 |
| 9 | 3590 | 3206 | 3087 | 2980 | 2861 | 2706 |  | 1902 | 407 | 201 | 3389 |  | 1802 | 400 | 216 | 3374 |
| 10 | 3520 | 3070 | 2970 | 2842 | 2712 | 2571 |  | 1814 | 395 | 191 | 3329 |  | 1681 | 251 | 131 | 3389 |
| Total | 48833 | 42605 | 40951 | 39421 | 37824 | 36050 |  | 23836 | 4277 | 2070 | 46763 |  | 23813 | 4257 | 2121 | 46707 |

**Table S4.** Markers distribution in the different subsets of markers, which were selected based on the two methods, linkage disequilibrium (LD) between markers and significant markers, in rice for two traits including panicles per plant (PPP) and seeds per plant (SPP).

|  |  |  | **LD** |  |  |  |  |  |  | **PPP** |  |  |  | **SPP** |  |  |
| --- | --- | --- | --- | --- | --- | --- | --- | --- | --- | --- | --- | --- | --- | --- | --- | --- |
| **CHR** | **Complete** | **LD_90** | **LD_80** | **LD_70** | **LD_60** | **LD_50** |  | **SNP_5** | **SNP_1** | **SNP_05** | **SNP_NS** |  | **SNP_5** | **SNP_1** | **SNP_05** | **SNP_NS** |
| 1 | 6125 | 4894 | 4675 | 4497 | 4246 | 3944 |  | 3205 | 490 | 212 | 5909 |  | 3292 | 660 | 306 | 5819 |
| 2 | 3629 | 2935 | 2722 | 2573 | 2418 | 2239 |  | 1260 | 144 | 67 | 3562 |  | 1785 | 204 | 71 | 3558 |
| 3 | 4118 | 3210 | 3035 | 2863 | 2686 | 2439 |  | 1310 | 46 | 13 | 4105 |  | 1732 | 125 | 43 | 4075 |
| 4 | 2729 | 2222 | 2095 | 1975 | 1825 | 1703 |  | 1232 | 244 | 139 | 2590 |  | 1453 | 192 | 91 | 2637 |
| 5 | 2702 | 2112 | 1966 | 1845 | 1720 | 1570 |  | 756 | 38 | 16 | 2686 |  | 1281 | 193 | 32 | 2670 |
| 6 | 3066 | 2610 | 2464 | 2332 | 2172 | 1992 |  | 1069 | 75 | 24 | 3041 |  | 1431 | 322 | 225 | 2841 |
| 7 | 1976 | 1588 | 1478 | 1377 | 1294 | 1162 |  | 605 | 45 | 11 | 1965 |  | 692 | 57 | 25 | 1951 |
| 8 | 2129 | 1769 | 1700 | 1627 | 1544 | 1443 |  | 840 | 70 | 24 | 2105 |  | 863 | 79 | 20 | 2109 |
| 9 | 1878 | 1571 | 1479 | 1405 | 1319 | 1218 |  | 636 | 39 | 12 | 1866 |  | 698 | 58 | 13 | 1865 |
| 10 | 1673 | 1399 | 1323 | 1272 | 1223 | 1169 |  | 711 | 88 | 48 | 1625 |  | 665 | 41 | 5 | 1668 |
| 11 | 2793 | 2374 | 2248 | 2140 | 2011 | 1867 |  | 1115 | 166 | 70 | 2723 |  | 1339 | 305 | 161 | 2632 |
| 12 | 2030 | 1706 | 1623 | 1531 | 1452 | 1361 |  | 791 | 109 | 38 | 1992 |  | 752 | 101 | 51 | 1979 |
| Total | 34848 | 28390 | 26808 | 25437 | 23910 | 22107 |  | 13530 | 1554 | 674 | 34169 |  | 15983 | 2337 | 1043 | 33804 |

**Table S5.** Correlations of narrow sense heritabilities and prediction accuracies of 11 different genomic prediction models for canopy wilting (CW) carbon isotope ratio ($\delta$^13^C), seeds per plant (SPP), panicles per plant (PPP), and days to tasseling (DT) and ear height (EH) at different training-to-testing proportions (90/10%, 70/30%, & 50/50%).

| **Soybean** |  |  |  | **CW** |  |  |  | $\boldsymbol{\delta}$**^13^C** |  |
| --- | --- | --- | --- | --- | --- | --- | --- | --- | --- |
|  |  | **90%** |  | **70%** | **50%** |  | **90%** | **70%** | **50%** |
|  | **GEBV_BayesA** | 0.29 |  | 0.41 | 0.45 |  | 0.46 | 0.72 | 0.56 |
|  | **GEBV_BayesB** | 0.5 |  | 0.36 | 0.46 |  | 0.62 | 0.69 | 0.58 |
|  | **GEBV_BayesC** | 0.11 |  | 0.44 | 0.43 |  | -0.14 | 0.72 | 0.57 |
|  | **GEBV_BL** | 0.04 |  | 0.4 | 0.41 |  | 0.53 | 0.68 | 0.58 |
|  | **GEBV_BLBL** | 0.04 |  | 0.31 | 0.39 |  | -0.2 | 0.67 | 0.57 |
|  | **GEBV_BLRR** | -0.25 |  | 0.33 | 0.47 |  | -0.17 | 0.68 | 0.59 |
|  | **GEBV_BRR** | -0.15 |  | 0.35 | 0.47 |  | 0.37 | 0.75 | 0.58 |
|  | **GEBV_NR** | -0.45 |  | 0.28 | 0.43 |  | 0.47 | 0.69 | 0.56 |
|  | **GEBV_RKHS** | -0.1 |  | 0.36 | 0.44 |  | 0.44 | 0.71 | 0.59 |
|  | **GEBV_rrBLUP** | 0.03 |  | 0.28 | 0.43 |  | 0.47 | 0.69 | 0.56 |
|  | **GBLUP** | 0.07 |  | 0.69 | 0.7 |  | 0.95 | 0.91 | 0.82 |
| **Rice** |  |  |  | **SPP** |  |  |  | **PPP** |  |
|  |  | **90%** |  | **70%** | **50%** |  | **90%** | **70%** | **50%** |
|  | **GEBV_BayesA** | 0.98 |  | 0.99 | 0.9 |  | 0.91 | 0.43 | 0.91 |
|  | **GEBV_BayesB** | 0.97 |  | 0.96 | 0.9 |  | 0.92 | 0.43 | 0.93 |
|  | **GEBV_BayesC** | 0.98 |  | 0.96 | 0.92 |  | 0.92 | 0.43 | 0.92 |
|  | **GEBV_BL** | 0.96 |  | 0.96 | 0.87 |  | 0.9 | 0.47 | 0.89 |
|  | **GEBV_BLBL** | 0.98 |  | 0.97 | 0.93 |  | 0.9 | 0.48 | 0.93 |
|  | **GEBV_BLRR** | 0.97 |  | 0.91 | 0.82 |  | 0.89 | 0.5 | 0.91 |
|  | **GEBV_BRR** | 0.98 |  | 0.96 | 0.9 |  | 0.9 | 0.47 | 0.92 |
|  | **GEBV_NR** | 0.97 |  | 0.99 | 0.91 |  | 0.64 | 0.51 | 0.85 |
|  | **GEBV_RKHS** | 0.97 |  | 0.97 | 0.87 |  | 0.65 | 0.45 | 0.79 |
|  | **GEBV_rrBLUP** | 0.97 |  | 0.98 | 0.91 |  | 0.64 | 0.51 | 0.85 |
|  | **GBLUP** | 0.96 |  | 0.99 | 0.94 |  | 0.89 | 0.48 | 0.92 |
| **Maize** |  |  |  | **DT** |  |  |  | **EH** |  |
|  | **Models** | **90%** |  | **70%** | **50%** |  | **90%** | **70%** | **50%** |
|  | **GEBV_BayesA** | 0.58 |  | 0.97 | 0.57 |  | 0.98 | 0.95 | 0.76 |
|  | **GEBV_BayesB** | 0.5 |  | 0.98 | 0.51 |  | 0.93 | 0.9 | 0.76 |
|  | **GEBV_BayesC** | 0.58 |  | 0.97 | 0.55 |  | 0.97 | 0.95 | 0.78 |
|  | **GEBV_BL** | 0.43 |  | 0.98 | 0.52 |  | 0.98 | 0.94 | 0.75 |
|  | **GEBV_BLBL** | 0.43 |  | 0.97 | 0.66 |  | 0.94 | 0.95 | 0.77 |
|  | **GEBV_BLRR** | 0.26 |  | 0.97 | 0.39 |  | 0.99 | 0.94 | 0.73 |
|  | **GEBV_BRR** | 0.81 |  | 0.97 | 0.57 |  | 0.98 | 0.93 | 0.83 |
|  | **GEBV_NR** | 0.9 |  | 1 | 0.7 |  | 0.63 | 0.51 | 0.2 |
|  | **GEBV_RKHS** | 0.44 |  | 0.97 | 0.59 |  | 0.94 | 0.93 | 0.79 |
|  | **GEBV_rrBLUP** | 0.9 |  | 0.98 | 0.7 |  | 0.63 | 0.51 | 0.2 |
|  | **GBLUP** | 0.91 |  | 0.98 | 0.9 |  | 1 | 0.93 | 0.8 |

**Table S6.** Prediction accuracy of genomic models for canopy wilting (CW) and carbon isotope ratio ($\delta$^13^C) in soybean, and seeds per plant (SPP) and panicles per plant (PPP) in rice, and days to tasseling (DT) and ear height (EH) in maize using different subsets of markers at the 90/10% training-to-testing proportion.

| **Trait** | **Marker Set** | **GEBV_BayesA** | **GEBV_BayesB** | **GEBV_BayesC** | **GEBV_BL** | **GEBV_BLBL** | **GEBV_BLRR** | **GEBV_BRR** | **GEBV_NR** | **GEBV_RKHS** | **GEBV_rrBLUP** | **GBLUP** |
| --- | --- | --- | --- | --- | --- | --- | --- | --- | --- | --- | --- | --- |
|  |  |  |  |  |  | **Soybean** |  |  |  |  |  |  |
| **CW** | Complete | 0.52 | 0.48 | 0.49 | 0.52 | 0.5 | 0.51 | 0.51 | 0.51 | 0.5 | 0.48 | 0.48 |
|  | SNP_5 | 0.53 | 0.56 | 0.55 | 0.54 | 0.54 | 0.53 | 0.53 | 0.52 | 0.53 | 0.52 | 0.52 |
|  | SNP_1 | 0.58 | 0.59 | 0.51 | 0.5 | 0.51 | 0.52 | 0.52 | 0.52 | 0.52 | 0.52 | 0.51 |
|  | SNP_05 | 0.55 | 0.6 | 0.53 | 0.53 | 0.52 | 0.47 | 0.49 | 0.49 | 0.49 | 0.49 | 0.49 |
|  | SNP_NS | 0.4 | 0.41 | 0.41 | 0.39 | 0.33 | 0.41 | 0.42 | 0.38 | 0.42 | 0.38 | 0.52 |
|  | LD_90 | 0.53 | 0.54 | 0.53 | 0.53 | 0.5 | 0.55 | 0.53 | 0.51 | 0.53 | 0.51 | 0.51 |
|  | LD_80 | 0.5 | 0.52 | 0.5 | 0.48 | 0.51 | 0.55 | 0.51 | 0.5 | 0.51 | 0.5 | 0.51 |
|  | LD_70 | 0.51 | 0.54 | 0.5 | 0.57 | 0.5 | 0.51 | 0.5 | 0.51 | 0.52 | 0.51 | 0.51 |
|  | LD_60 | 0.54 | 0.57 | 0.54 | 0.49 | 0.5 | 0.54 | 0.52 | 0.51 | 0.53 | 0.51 | 0.52 |
|  | LD_50 | 0.5 | 0.52 | 0.48 | 0.5 | 0.53 | 0.49 | 0.51 | 0.49 | 0.51 | 0.49 | 0.5 |
|  |  |  |  |  |  |  |  |  |  |  |  |  |
| $\boldsymbol{\delta}$**^13^C** | Complete | 0.4 | 0.41 | 0.4 | 0.4 | 0..41 | 0..40 | 0.41 | 0.41 | 0.42 | 0.41 | 0.4 |
|  | SNP_5 | 0.52 | 0.51 | 0.48 | 0.51 | 0.51 | 0.51 | 0.51 | 0.49 | 0.51 | 0.49 | 0.48 |
|  | SNP_1 | 0.5 | 0.52 | 0.48 | 0.48 | 0.47 | 0.47 | 0.47 | 0.48 | 0.48 | 0.48 | 0.48 |
|  | SNP_05 | 0.54 | 0.61 | 0.58 | 0.54 | 0.53 | 0.51 | 0.5 | 0.5 | 0.51 | 0.5 | 0.5 |
|  | SNP_NS | 0.36 | 0.38 | 0.37 | 0.37 | 0.36 | 0.39 | 0.39 | 0.38 | 0.38 | 0.38 | 0.48 |
|  | LD_90 | 0.44 | 0.45 | 0.45 | 0.43 | 0.46 | 0.44 | 0.44 | 0.44 | 0.45 | 0.44 | 0.43 |
|  | LD_80 | 0.45 | 0.45 | 0.46 | 0.45 | 0.46 | 0.47 | 0.46 | 0.44 | 0.45 | 0.44 | 0.43 |
|  | LD_70 | 0.46 | 0.47 | 0.45 | 0.46 | 0.45 | 0.44 | 0.46 | 0.45 | 0.46 | 0.45 | 0.43 |
|  | LD_60 | 0.47 | 0.46 | 0.45 | 0.44 | 0.45 | 0.46 | 0.46 | 0.45 | 0.45 | 0.45 | 0.44 |
|  | LD_50 | 0.47 | 0.46 | 0.48 | 0.46 | 0.45 | 0.43 | 0.47 | 0.45 | 0.46 | 0.45 | 0.44 |
|  |  |  |  |  |  |  |  |  |  |  |  |  |
|  |  |  |  |  |  | **Table S6 (continued)**  **Rice** |  |  |  |  |  |  |
| **SPP** | Complete | 0.57 | 0.52 | 0.53 | 0.46 | 0.6 | 0.52 | 0.53 | 0.52 | 0.53 | 0.51 | 0.54 |
|  | SNP_5 | 0.69 | 0.68 | 0.66 | 0.62 | 0.69 | 0.64 | 0.67 | 0.66 | 0.65 | 0.66 | 0.68 |
|  | SNP_1 | 0.72 | 0.74 | 0.7 | 0.7 | 0.69 | 0.67 | 0.69 | 0.68 | 0.69 | 0.68 | 0.69 |
|  | SNP_05 | 0.7 | 0.75 | 0.71 | 0.69 | 0.7 | 0.63 | 0.64 | 0.64 | 0.66 | 0.64 | 0.64 |
|  | SNP_NS | 0.5 | 0.51 | 0.47 | 0.46 | 0.55 | 0.49 | 0.47 | 0.44 | 0.51 | 0.44 | 0.47 |
|  | LD_90 | 0.57 | 0.53 | 0.53 | 0.58 | 0.61 | 0.51 | 0.53 | 0.53 | 0.54 | 0.53 | 0.55 |
|  | LD_80 | 0.6 | 0.57 | 0.51 | 0.55 | 0.62 | 0.54 | 0.53 | 0.53 | 0.55 | 0.53 | 0.55 |
|  | LD_70 | 0.56 | 0.55 | 0.55 | 0.52 | 0.61 | 0.53 | 0.54 | 0.53 | 0.53 | 0.53 | 0.56 |
|  | LD_60 | 0.56 | 0.54 | 0.54 | 0.54 | 0.61 | 0.55 | 0.54 | 0.52 | 0.55 | 0.52 | 0.55 |
|  | LD_50 | 0.59 | 0.55 | 0.55 | 0.52 | 0.59 | 0.57 | 0.54 | 0.53 | 0.53 | 0.52 | 0.55 |
|  |  |  |  |  |  |  |  |  |  |  |  |  |
| **PPP** | Complete | 0.7 | 0.7 | 0.7 | 0.7 | 0.69 | 0.7 | 0.7 | 0.7 | 0.7 | 0.7 | 0.7 |
|  | SNP_5 | 0.76 | 0.77 | 0.76 | 0.76 | 0.75 | 0.75 | 0.76 | 0.75 | 0.74 | 0.75 | 0.75 |
|  | SNP_1 | 0.82 | 0.82 | 0.82 | 0.82 | 0.81 | 0.81 | 0.81 | 0.71 | 0.72 | 0.71 | 0.8 |
|  | SNP_05 | 0.84 | 0.83 | 0.84 | 0.84 | 0.84 | 0.84 | 0.84 | 0.83 | 0.84 | 0.83 | 0.83 |
|  | SNP_NS | 0.68 | 0.68 | 0.68 | 0.68 | 0.67 | 0.68 | 0.67 | 0.68 | 0.69 | 0.68 | 0.68 |
|  | LD_90 | 0.7 | 0.69 | 0.7 | 0.7 | 0.69 | 0.69 | 0.7 | 0.69 | 0.7 | 0.69 | 0.7 |
|  | LD_80 | 0.7 | 0.71 | 0.7 | 0.7 | 0.69 | 0.7 | 0.7 | 0.69 | 0.69 | 0.69 | 0.69 |
|  | LD_70 | 0.7 | 0.7 | 0.7 | 0.69 | 0.69 | 0.7 | 0.69 | 0.7 | 0.7 | 0.7 | 0.7 |
|  | LD_60 | 0.7 | 0.7 | 0.7 | 0.69 | 0.69 | 0.69 | 0.7 | 0.7 | 0.7 | 0.7 | 0.7 |
|  | LD_50 | 0.7 | 0.7 | 0.69 | 0.69 | 0.7 | 0.7 | 0.7 | 0.7 | 0.7 | 0.7 | 0.7 |
|  |  |  |  |  |  |  |  |  |  |  |  |  |
|  |  |  |  |  |  | **Table S6 (continued)**  **Mazie** |  |  |  |  |  |  |
| **DP** | Complete | 0.67 | 0.67 | 0.67 | 0.67 | 0.7 | 0.67 | 0.66 | 0.65 | 0.68 | 0.65 | 0.65 |
|  | SNP_5 | 0.68 | 0.69 | 0.68 | 0.69 | 0.65 | 0.66 | 0.68 | 0.66 | 0.68 | 0.66 | 0.65 |
|  | SNP_1 | 0.68 | 0.68 | 0.69 | 0.69 | 0.68 | 0.66 | 0.68 | 0.67 | 0.69 | 0.67 | 0.65 |
|  | SNP_05 | 0.68 | 0.73 | 0.69 | 0.68 | 0.67 | 0.66 | 0.67 | 0.66 | 0.73 | 0.66 | 0.65 |
|  | SNP_NS | 0.67 | 0.67 | 0.67 | 0.68 | 0.62 | 0.66 | 0.66 | 0.63 | 0.67 | 0.63 | 0.64 |
|  | LD_90 | 0.66 | 0.68 | 0.67 | 0.69 | 0.62 | 0.64 | 0.67 | 0.65 | 0.67 | 0.65 | 0.65 |
|  | LD_80 | 0.67 | 0.67 | 0.67 | 0.68 | 0.64 | 0.65 | 0.67 | 0.65 | 0.66 | 0.65 | 0.65 |
|  | LD_70 | 0.66 | 0.67 | 0.68 | 0.67 | 0.67 | 0.67 | 0.66 | 0.64 | 0.67 | 0.64 | 0.65 |
|  | LD_60 | 0.67 | 0.67 | 0.67 | 0.69 | 0.64 | 0.63 | 0.67 | 0.65 | 0.67 | 0.65 | 0.65 |
|  | LD_50 | 0.66 | 0.68 | 0.66 | 0.69 | 0.63 | 0.64 | 0.67 | 0.64 | 0.67 | 0.64 | 0.65 |
|  |  |  |  |  |  |  |  |  |  |  |  |  |
| **EH** | Complete | 0.68 | 0.7 | 0.69 | 0.68 | 0.69 | 0.7 | 0.68 | 0.69 | 0.69 | 0.69 | 0.69 |
|  | SNP_5 | 0.81 | 0.8 | 0.82 | 0.83 | 0.83 | 0.8 | 0.79 | 0.84 | 0.8 | 0.84 | 0.79 |
|  | SNP_1 | 0.86 | 0.85 | 0.85 | 0.84 | 0.85 | 0.86 | 0.84 | 0.84 | 0.85 | 0.84 | 0.84 |
|  | SNP_05 | 0.86 | 0.91 | 0.86 | 0.83 | 0.85 | 0.84 | 0.83 | 0.8 | 0.9 | 0.8 | 0.81 |
|  | SNP_NS | 0.59 | 0.59 | 0.62 | 0.62 | 0.57 | 0.64 | 0.62 | 0.8 | 0.62 | 0.8 | 0.61 |
|  | LD_90 | 0.7 | 0.7 | 0.69 | 0.7 | 0.66 | 0.69 | 0.69 | 0.7 | 0.7 | 0.7 | 0.7 |
|  | LD_80 | 0.7 | 0.7 | 0.71 | 0.68 | 0.76 | 0.7 | 0.71 | 0.7 | 0.7 | 0.7 | 0.7 |
|  | LD_70 | 0.7 | 0.69 | 0.7 | 0.69 | 0.66 | 0.69 | 0.7 | 0.7 | 0.7 | 0.7 | 0.69 |
|  | LD_60 | 0.7 | 0.68 | 0.68 | 0.71 | 0.72 | 0.71 | 0.7 | 0.7 | 0.7 | 0.7 | 0.7 |
|  | LD_50 | 0.7 | 0.68 | 0.72 | 0.7 | 0.66 | 0.71 | 0.68 | 0.7 | 0.7 | 0.7 | 0.7 |

**Table S7.** Prediction accuracy of genomic models for canopy wilting (CW) and carbon isotope ratio ($\delta$^13^C) in soybean, and seeds per plant (SPP) and panicles per plant (PPP) in rice, and days to tasseling (DT) and ear height (EH) in maize using different subsets of markers at the 70/30% training-to-testing proportion..

| **Trait** | **Marker Set** | **GEBV_BayesA** | **GEBV_BayesB** | **GEBV_BayesC** | **GEBV_BL** | **GEBV_BLBL** | **GEBV_BLRR** | **GEBV_BRR** | **GEBV_NR** | **GEBV_RKHS** | **GEBV_rrBLUP** | **GBLUP** |
| --- | --- | --- | --- | --- | --- | --- | --- | --- | --- | --- | --- | --- |
|  |  |  |  |  |  | **Soybean** |  |  |  |  |  |  |
| **CW** | Complete | 0.55 | 0.57 | 0.54 | 0.55 | 0.56 | 0.55 | 0.56 | 0.56 | 0.56 | 0.57 | 0.56 |
|  | SNP_5 | 0.66 | 0.65 | 0.65 | 0.63 | 0.67 | 0.62 | 0.65 | 0.65 | 0.65 | 0.65 | 0.65 |
|  | SNP_1 | 0.66 | 0.67 | 0.66 | 0.65 | 0.67 | 0.67 | 0.65 | 0.65 | 0.65 | 0.65 | 0.65 |
|  | SNP_05 | 0.66 | 0.68 | 0.66 | 0.66 | 0.66 | 0.63 | 0.63 | 0.63 | 0.63 | 0.63 | 0.63 |
|  | SNP_NS | 0.47 | 0.45 | 0.46 | 0.46 | 0.41 | 0.44 | 0.46 | 0.45 | 0.46 | 0.45 | 0.65 |
|  | LD_90 | 0.55 | 0.57 | 0.56 | 0.55 | 0.54 | 0.47 | 0.55 | 0.55 | 0.54 | 0.55 | 0.55 |
|  | LD_80 | 0.55 | 0.55 | 0.53 | 0.52 | 0.54 | 0.52 | 0.54 | 0.55 | 0.55 | 0.55 | 0.55 |
|  | LD_70 | 0.55 | 0.55 | 0.53 | 0.54 | 0.55 | 0.45 | 0.53 | 0.55 | 0.55 | 0.55 | 0.55 |
|  | LD_60 | 0.58 | 0.56 | 0.56 | 0.55 | 0.57 | 0.54 | 0.56 | 0.56 | 0.54 | 0.56 | 0.55 |
|  | LD_50 | 0.57 | 0.56 | 0.52 | 0.54 | 0.53 | 0.54 | 0.55 | 0.55 | 0.54 | 0.55 | 0.55 |
|  |  |  |  |  |  |  |  |  |  |  |  |  |
| $\boldsymbol{\delta}$**^13^C** | Complete | 0.24 | 0.25 | 0.25 | 0.27 | 0.22 | 0.27 | 0.25 | 0.24 | 0.24 | 0.24 | 0.25 |
|  | SNP_5 | 0.34 | 0.34 | 0.35 | 0.35 | 0.35 | 0.36 | 0.35 | 0.33 | 0.35 | 0.33 | 0.33 |
|  | SNP_1 | 0.42 | 0.45 | 0.42 | 0.41 | 0.4 | 0.4 | 0.41 | 0.39 | 0.4 | 0.39 | 0.39 |
|  | SNP_05 | 0.49 | 0.51 | 0.52 | 0.49 | 0.49 | 0.47 | 0.47 | 0.47 | 0.47 | 0.47 | 0.46 |
|  | SNP_NS | 0.21 | 0.21 | 0.23 | 0.23 | 0.16 | 0.23 | 0.24 | 0.21 | 0.22 | 0.21 | 0.33 |
|  | LD_90 | 0.24 | 0.24 | 0.23 | 0.28 | 0.2 | 0.26 | 0.24 | 0.24 | 0.24 | 0.24 | 0.24 |
|  | LD_80 | 0.25 | 0.25 | 0.25 | 0.25 | 0.21 | 0.27 | 0.27 | 0.24 | 0.25 | 0.24 | 0.25 |
|  | LD_70 | 0.23 | 0.23 | 0.25 | 0.26 | 0.22 | 0.25 | 0.26 | 0.24 | 0.26 | 0.24 | 0.25 |
|  | LD_60 | 0.24 | 0.26 | 0.27 | 0.27 | 0.21 | 0.29 | 0.25 | 0.25 | 0.26 | 0.25 | 0.26 |
|  | LD_50 | 0.25 | 0.26 | 0.27 | 0.28 | 0.26 | 0.28 | 0.26 | 0.26 | 0.27 | 0.26 | 0.27 |
|  |  |  |  |  |  |  |  |  |  |  |  |  |
|  |  |  |  |  |  | **Table S7. (Continued)**  **Rice** |  |  |  |  |  |  |
| **SPP** | Complete | 0.35 | 0.33 | 0.32 | 0.33 | 0.47 | 0.35 | 0.33 | 0.3 | 0.32 | 0.3 | 0.3 |
|  | SNP_5 | 0.5 | 0.6 | 0.55 | 0.56 | 0.68 | 0.44 | 0.57 | 0.42 | 0.42 | 0.42 | 0.42 |
|  | SNP_1 | 0.6 | 0.59 | 0.57 | 0.57 | 0.56 | 0.56 | 0.57 | 0.56 | 0.56 | 0.56 | 0.53 |
|  | SNP_05 | 0.64 | 0.69 | 0.65 | 0.63 | 0.62 | 0.6 | 0.59 | 0.6 | 0.59 | 0.6 | 0.57 |
|  | SNP_NS | 0.3 | 0.26 | 0.27 | 0.28 | 0.38 | 0.26 | 0.28 | 0.26 | 0.31 | 0.26 | 0.25 |
|  | LD_90 | 0.37 | 0.36 | 0.35 | 0.33 | 0.46 | 0.25 | 0.36 | 0.32 | 0.34 | 0.32 | 0.32 |
|  | LD_80 | 0.35 | 0.35 | 0.31 | 0.35 | 0.45 | 0.29 | 0.35 | 0.32 | 0.33 | 0.32 | 0.31 |
|  | LD_70 | 0.35 | 0.35 | 0.36 | 0.37 | 0.47 | 0.33 | 0.35 | 0.32 | 0.34 | 0.32 | 0.31 |
|  | LD_60 | 0.34 | 0.33 | 0.34 | 0.29 | 0.43 | 0.31 | 0.32 | 0.31 | 0.33 | 0.31 | 0.31 |
|  | LD_50 | 0.37 | 0.35 | 0.36 | 0.33 | 0.44 | 0.3 | 0.31 | 0.32 | 0.34 | 0.32 | 0.32 |
|  |  |  |  |  |  |  |  |  |  |  |  |  |
| **PPP** | Complete | 0.66 | 0.66 | 0.66 | 0.64 | 0.66 | 0.65 | 0.65 | 0.65 | 0.65 | 0.65 | 0.64 |
|  | SNP_5 | 0.7 | 0.7 | 0.7 | 0.69 | 0.7 | 0.7 | 0.7 | 0.7 | 0.69 | 0.7 | 0.69 |
|  | SNP_1 | 0.77 | 0.78 | 0.78 | 0.77 | 0.77 | 0.76 | 0.76 | 0.66 | 0.66 | 0.66 | 0.76 |
|  | SNP_05 | 0.78 | 0.79 | 0.79 | 0.78 | 0.78 | 0.77 | 0.77 | 0.78 | 0.77 | 0.78 | 0.78 |
|  | SNP_NS | 0.64 | 0.64 | 0.64 | 0.63 | 0.64 | 0.64 | 0.64 | 0.63 | 0.65 | 0.63 | 0.63 |
|  | LD_90 | 0.66 | 0.67 | 0.66 | 0.65 | 0.66 | 0.66 | 0.66 | 0.66 | 0.65 | 0.66 | 0.65 |
|  | LD_80 | 0.66 | 0.67 | 0.66 | 0.65 | 0.67 | 0.66 | 0.67 | 0.66 | 0.66 | 0.66 | 0.65 |
|  | LD_70 | 0.67 | 0.67 | 0.66 | 0.65 | 0.67 | 0.66 | 0.66 | 0.66 | 0.65 | 0.66 | 0.65 |
|  | LD_60 | 0.67 | 0.66 | 0.66 | 0.65 | 0.67 | 0.66 | 0.66 | 0.66 | 0.66 | 0.66 | 0.66 |
|  | LD_50 | 0.66 | 0.67 | 0.66 | 0.65 | 0.67 | 0.66 | 0.66 | 0.66 | 0.66 | 0.66 | 0.66 |
|  |  |  |  |  |  |  |  |  |  |  |  |  |
|  |  |  |  |  |  | **Table S7. (Continued)**  **Maize** |  |  |  |  |  |  |
| **DP** | Complete | 0.59 | 0.58 | 0.59 | 0.58 | 0.6 | 0.59 | 0.59 | 0.59 | 0.59 | 0.59 | 0.59 |
|  | SNP_5 | 0.64 | 0.64 | 0.65 | 0.64 | 0.66 | 0.64 | 0.64 | 0.64 | 0.65 | 0.64 | 0.64 |
|  | SNP_1 | 0.72 | 0.73 | 0.72 | 0.72 | 0.72 | 0.72 | 0.71 | 0.72 | 0.72 | 0.72 | 0.71 |
|  | SNP_05 | 0.71 | 0.73 | 0.71 | 0.71 | 0.71 | 0.7 | 0.7 | 0.73 | 0.71 | 0.73 | 0.72 |
|  | SNP_NS | 0.56 | 0.56 | 0.56 | 0.55 | 0.55 | 0.56 | 0.56 | 0.56 | 0.56 | 0.56 | 0.56 |
|  | LD_90 | 0.6 | 0.6 | 0.58 | 0.58 | 0.59 | 0.58 | 0.59 | 0.59 | 0.59 | 0.59 | 0.59 |
|  | LD_80 | 0.58 | 0.58 | 0.59 | 0.59 | 0.57 | 0.59 | 0.58 | 0.59 | 0.59 | 0.59 | 0.59 |
|  | LD_70 | 0.59 | 0.58 | 0.58 | 0.58 | 0.58 | 0.59 | 0.58 | 0.59 | 0.59 | 0.59 | 0.59 |
|  | LD_60 | 0.59 | 0.6 | 0.59 | 0.59 | 0.56 | 0.6 | 0.59 | 0.59 | 0.59 | 0.59 | 0.59 |
|  | LD_50 | 0.59 | 0.59 | 0.59 | 0.59 | 0.58 | 0.6 | 0.6 | 0.6 | 0.59 | 0.6 | 0.59 |
|  |  |  |  |  |  |  |  |  |  |  |  |  |
| **EH** | Complete | 0.55 | 0.55 | 0.55 | 0.57 | 0.54 | 0.55 | 0.54 | 0.55 | 0.55 | 0.55 | 0.54 |
|  | SNP_5 | 0.68 | 0.67 | 0.69 | 0.66 | 0.7 | 0.69 | 0.67 | 0.76 | 0.67 | 0.76 | 0.66 |
|  | SNP_1 | 0.75 | 0.74 | 0.75 | 0.73 | 0.77 | 0.74 | 0.74 | 0.76 | 0.75 | 0.76 | 0.73 |
|  | SNP_05 | 0.76 | 0.79 | 0.75 | 0.74 | 0.78 | 0.75 | 0.74 | 0.75 | 0.76 | 0.75 | 0.73 |
|  | SNP_NS | 0.47 | 0.49 | 0.48 | 0.48 | 0.44 | 0.49 | 0.5 | 0.75 | 0.49 | 0.75 | 0.49 |
|  | LD_90 | 0.56 | 0.54 | 0.54 | 0.55 | 0.53 | 0.53 | 0.54 | 0.56 | 0.55 | 0.56 | 0.54 |
|  | LD_80 | 0.56 | 0.57 | 0.54 | 0.55 | 0.53 | 0.54 | 0.54 | 0.56 | 0.55 | 0.56 | 0.55 |
|  | LD_70 | 0.55 | 0.55 | 0.55 | 0.55 | 0.51 | 0.56 | 0.56 | 0.56 | 0.54 | 0.56 | 0.54 |
|  | LD_60 | 0.55 | 0.55 | 0.56 | 0.56 | 0.55 | 0.55 | 0.54 | 0.56 | 0.55 | 0.56 | 0.55 |
|  | LD_50 | 0.55 | 0.55 | 0.54 | 0.55 | 0.49 | 0.54 | 0.54 | 0.56 | 0.55 | 0.56 | 0.54 |

**Table S8.** Prediction accuracy of genomic models for canopy wilting (CW) and carbon isotope ratio ($\delta$^13^C) in soybean, and seeds per plant (SPP) and panicles per plant (PPP) in rice, and days to tasseling (DT) and ear height (EH) in maize using different subsets of markers at the 50/50% training-to-testing proportion.

| **Trait** | **Marker Set** | **GEBV_BayesA** | **GEBV_BayesB** | **GEBV_BayesC** | **GEBV_BL** | **GEBV_BLBL** | **GEBV_BLRR** | **GEBV_BRR** | **GEBV_NR** | **GEBV_RKHS** | **GEBV_rrBLUP** | **GBLUP** |
| --- | --- | --- | --- | --- | --- | --- | --- | --- | --- | --- | --- | --- |
|  |  |  |  |  |  | **Soybean** |  |  |  |  |  |  |
| **CW** | Complete | 0.26 | 0.26 | 0.26 | 0.28 | 0.25 | 0.3 | 0.27 | 0.24 | 0.25 | 0.24 | 0.25 |
|  | SNP_5 | 0.41 | 0.41 | 0.41 | 0.41 | 0.4 | 0.41 | 0.41 | 0.4 | 0.41 | 0.4 | 0.4 |
|  | SNP_1 | 0.49 | 0.5 | 0.48 | 0.47 | 0.49 | 0.47 | 0.48 | 0.47 | 0.47 | 0.47 | 0.47 |
|  | SNP_05 | 0.55 | 0.57 | 0.54 | 0.54 | 0.53 | 0.52 | 0.52 | 0.53 | 0.52 | 0.53 | 0.52 |
|  | SNP_NS | 0.18 | 0.18 | 0.2 | 0.18 | 0.12 | 0.2 | 0.18 | 0.15 | 0.19 | 0.15 | 0.4 |
|  | LD_90 | 0.27 | 0.28 | 0.3 | 0.3 | 0.26 | 0.28 | 0.28 | 0.26 | 0.29 | 0.26 | 0.27 |
|  | LD_80 | 0.27 | 0.29 | 0.27 | 0.29 | 0.29 | 0.27 | 0.28 | 0.26 | 0.28 | 0.26 | 0.26 |
|  | LD_70 | 0.28 | 0.29 | 0.28 | 0.28 | 0.28 | 0.27 | 0.28 | 0.26 | 0.28 | 0.26 | 0.26 |
|  | LD_60 | 0.3 | 0.28 | 0.3 | 0.3 | 0.27 | 0.3 | 0.29 | 0.28 | 0.3 | 0.28 | 0.29 |
|  | LD_50 | 0.29 | 0.29 | 0.3 | 0.3 | 0.3 | 0.29 | 0.28 | 0.28 | 0.3 | 0.28 | 0.29 |
|  |  |  |  |  |  |  |  |  |  |  |  |  |
| $\boldsymbol{\delta}$**^13^C** | Complete | 0.32 | 0.32 | 0.32 | 0.32 | 0.32 | 0.31 | 0.32 | 0.31 | 0.32 | 0.31 | 0.32 |
|  | SNP_5 | 0.41 | 0.42 | 0.42 | 0.43 | 0.42 | 0.41 | 0.43 | 0.41 | 0.41 | 0.41 | 0.41 |
|  | SNP_1 | 0.48 | 0.51 | 0.48 | 0.48 | 0.49 | 0.48 | 0.48 | 0.46 | 0.47 | 0.46 | 0.46 |
|  | SNP_05 | 0.54 | 0.54 | 0.53 | 0.53 | 0.53 | 0.52 | 0.52 | 0.51 | 0.51 | 0.51 | 0.51 |
|  | SNP_NS | 0.28 | 0.29 | 0.29 | 0.29 | 0.28 | 0.29 | 0.29 | 0.29 | 0.3 | 0.29 | 0.41 |
|  | LD_90 | 0.31 | 0.32 | 0.32 | 0.33 | 0.32 | 0.33 | 0.32 | 0.32 | 0.32 | 0.32 | 0.33 |
|  | LD_80 | 0.32 | 0.33 | 0.33 | 0.33 | 0.3 | 0.33 | 0.31 | 0.33 | 0.33 | 0.33 | 0.33 |
|  | LD_70 | 0.32 | 0.33 | 0.33 | 0.34 | 0.32 | 0.33 | 0.32 | 0.33 | 0.33 | 0.33 | 0.34 |
|  | LD_60 | 0.33 | 0.34 | 0.34 | 0.33 | 0.33 | 0.33 | 0.34 | 0.34 | 0.34 | 0.34 | 0.34 |
|  | LD_50 | 0.32 | 0.34 | 0.34 | 0.34 | 0.34 | 0.33 | 0.34 | 0.34 | 0.34 | 0.34 | 0.34 |
|  |  |  |  |  |  | **Table S8. (Continued)**  **Rice** |  |  |  |  |  |  |
| **SPP** | Complete | 0.3 | 0.3 | 0.28 | 0.27 | 0.3 | 0.25 | 0.27 | 0.24 | 0.28 | 0.24 | 0.19 |
|  | SNP_5 | 0.41 | 0.43 | 0.4 | 0.36 | 0.43 | 0.31 | 0.38 | 0.37 | 0.36 | 0.37 | 0.32 |
|  | SNP_1 | 0.51 | 0.56 | 0.51 | 0.5 | 0.51 | 0.5 | 0.5 | 0.49 | 0.49 | 0.49 | 0.41 |
|  | SNP_05 | 0.55 | 0.57 | 0.53 | 0.52 | 0.52 | 0.5 | 0.5 | 0.49 | 0.5 | 0.49 | 0.42 |
|  | SNP_NS | 0.23 | 0.25 | 0.23 | 0.22 | 0.24 | 0.18 | 0.22 | 0.19 | 0.26 | 0.18 | 0.14 |
|  | LD_90 | 0.29 | 0.28 | 0.28 | 0.24 | 0.31 | 0.26 | 0.27 | 0.25 | 0.28 | 0.25 | 0.19 |
|  | LD_80 | 0.31 | 0.32 | 0.28 | 0.28 | 0.3 | 0.19 | 0.27 | 0.25 | 0.29 | 0.25 | 0.19 |
|  | LD_70 | 0.29 | 0.28 | 0.28 | 0.27 | 0.33 | 0.21 | 0.29 | 0.26 | 0.28 | 0.25 | 0.2 |
|  | LD_60 | 0.3 | 0.31 | 0.29 | 0.3 | 0.3 | 0.25 | 0.28 | 0.25 | 0.28 | 0.25 | 0.19 |
|  | LD_50 | 0.3 | 0.25 | 0.26 | 0.26 | 0.32 | 0.28 | 0.28 | 0.26 | 0.28 | 0.26 | 0.2 |
|  |  |  |  |  |  |  |  |  |  |  |  |  |
| **PPP** | Complete | 0.69 | 0.7 | 0.69 | 0.7 | 0.69 | 0.7 | 0.69 | 0.69 | 0.69 | 0.69 | 0.69 |
|  | SNP_5 | 0.74 | 0.75 | 0.75 | 0.73 | 0.75 | 0.74 | 0.74 | 0.74 | 0.73 | 0.74 | 0.74 |
|  | SNP_1 | 0.8 | 0.8 | 0.81 | 0.8 | 0.8 | 0.8 | 0.8 | 0.72 | 0.71 | 0.72 | 0.8 |
|  | SNP_05 | 0.8 | 0.79 | 0.81 | 0.81 | 0.81 | 0.81 | 0.81 | 0.8 | 0.8 | 0.8 | 0.8 |
|  | SNP_NS | 0.68 | 0.68 | 0.68 | 0.69 | 0.67 | 0.69 | 0.68 | 0.68 | 0.69 | 0.68 | 0.68 |
|  | LD_90 | 0.7 | 0.7 | 0.7 | 0.7 | 0.69 | 0.7 | 0.7 | 0.7 | 0.69 | 0.7 | 0.7 |
|  | LD_80 | 0.7 | 0.7 | 0.7 | 0.7 | 0.69 | 0.7 | 0.7 | 0.7 | 0.7 | 0.7 | 0.7 |
|  | LD_70 | 0.7 | 0.7 | 0.7 | 0.7 | 0.7 | 0.7 | 0.7 | 0.7 | 0.7 | 0.7 | 0.7 |
|  | LD_60 | 0.7 | 0.7 | 0.7 | 0.7 | 0.69 | 0.7 | 0.7 | 0.7 | 0.7 | 0.7 | 0.7 |
|  | LD_50 | 0.7 | 0.71 | 0.7 | 0.7 | 0.69 | 0.7 | 0.7 | 0.7 | 0.7 | 0.7 | 0.7 |
|  |  |  |  |  |  | **Table S8. (Continued)**  **Maize** |  |  |  |  |  |  |
| **DP** | Complete | 0.69 | 0.69 | 0.69 | 0.68 | 0.69 | 0.63 | 0.69 | 0.7 | 0.69 | 0.7 | 0.72 |
|  | SNP_5 | 0.74 | 0.73 | 0.73 | 0.73 | 0.74 | 0.72 | 0.72 | 0.74 | 0.73 | 0.74 | 0.74 |
|  | SNP_1 | 0.76 | 0.76 | 0.76 | 0.75 | 0.77 | 0.76 | 0.76 | 0.76 | 0.76 | 0.76 | 0.76 |
|  | SNP_05 | 0.73 | 0.74 | 0.73 | 0.74 | 0.73 | 0.74 | 0.73 | 0.75 | 0.74 | 0.75 | 0.74 |
|  | SNP_NS | 0.68 | 0.68 | 0.68 | 0.67 | 0.67 | 0.67 | 0.67 | 0.67 | 0.67 | 0.67 | 0.67 |
|  | LD_90 | 0.71 | 0.7 | 0.7 | 0.67 | 0.71 | 0.72 | 0.69 | 0.7 | 0.7 | 0.7 | 0.72 |
|  | LD_80 | 0.7 | 0.7 | 0.7 | 0.69 | 0.7 | 0.66 | 0.7 | 0.71 | 0.7 | 0.71 | 0.72 |
|  | LD_70 | 0.7 | 0.7 | 0.69 | 0.68 | 0.7 | 0.69 | 0.7 | 0.71 | 0.7 | 0.71 | 0.72 |
|  | LD_60 | 0.71 | 0.7 | 0.7 | 0.69 | 0.71 | 0.67 | 0.7 | 0.71 | 0.7 | 0.71 | 0.72 |
|  | LD_50 | 0.7 | 0.7 | 0.69 | 0.69 | 0.71 | 0.71 | 0.7 | 0.71 | 0.7 | 0.71 | 0.72 |
|  |  |  |  |  |  |  |  |  |  |  |  |  |
| **EH** | Complete | 0.45 | 0.45 | 0.47 | 0.46 | 0.46 | 0.44 | 0.46 | 0.47 | 0.47 | 0.47 | 0.45 |
|  | SNP_5 | 0.54 | 0.56 | 0.55 | 0.54 | 0.54 | 0.54 | 0.55 | 0.59 | 0.55 | 0.59 | 0.52 |
|  | SNP_1 | 0.59 | 0.6 | 0.59 | 0.57 | 0.61 | 0.61 | 0.59 | 0.59 | 0.59 | 0.59 | 0.57 |
|  | SNP_05 | 0.59 | 0.61 | 0.59 | 0.58 | 0.6 | 0.6 | 0.59 | 0.59 | 0.6 | 0.59 | 0.56 |
|  | SNP_NS | 0.43 | 0.43 | 0.44 | 0.44 | 0.41 | 0.43 | 0.41 | 0.59 | 0.43 | 0.59 | 0.42 |
|  | LD_90 | 0.47 | 0.48 | 0.47 | 0.48 | 0.44 | 0.47 | 0.48 | 0.47 | 0.48 | 0.47 | 0.46 |
|  | LD_80 | 0.47 | 0.47 | 0.47 | 0.48 | 0.44 | 0.45 | 0.48 | 0.47 | 0.47 | 0.47 | 0.46 |
|  | LD_70 | 0.5 | 0.47 | 0.47 | 0.47 | 0.45 | 0.47 | 0.48 | 0.48 | 0.48 | 0.48 | 0.46 |
|  | LD_60 | 0.49 | 0.47 | 0.48 | 0.47 | 0.48 | 0.45 | 0.48 | 0.48 | 0.48 | 0.48 | 0.47 |
|  | LD_50 | 0.47 | 0.48 | 0.5 | 0.48 | 0.45 | 0.48 | 0.48 | 0.48 | 0.48 | 0.48 | 0.46 |
