# Supplementary figures and images for: Genomic prediction models for traits differing in heritability for soybean, rice, and maize
Source: BMC Plant Biol. 2022 Feb 26;22:87. doi: 10.1186/s12870-022-03479-y (PMC8881851; doi:10.1186/s12870-022-03479-y)

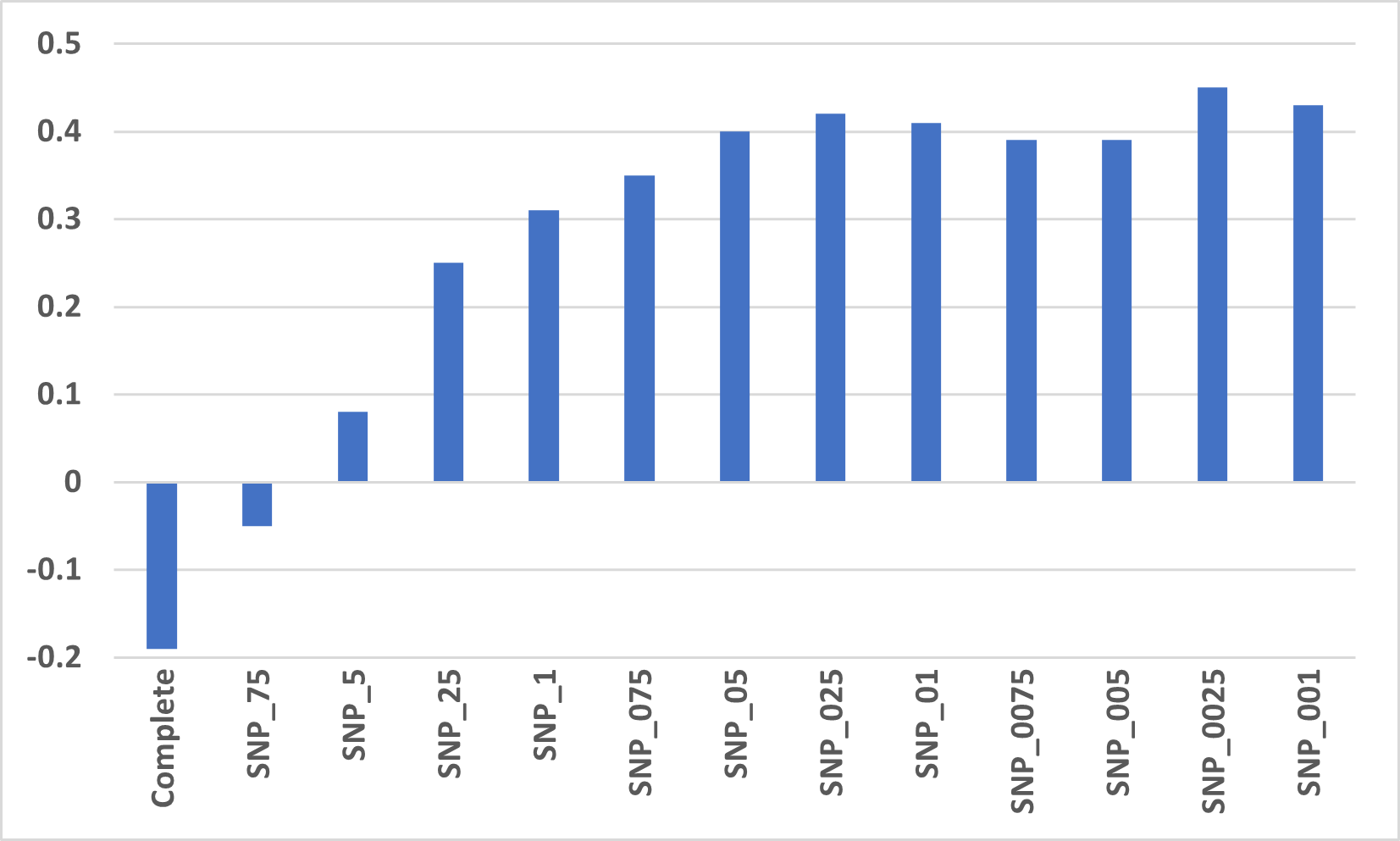

Supplement: Supplementary file 2 — Additional file 2: Fig. S1. Prediction accuracies for carbon isotope ratio (δ13) in soybean using BayesB model and a training-to-testing cross-validation proportion of 90:10%. Thirteen sets of marker subsets were selected based on the different significant levels including P < 0.001, P < 0.0025, P < 0.005, P < 0.0075, P < 0.01, P < 0.025, P < 0.05, P < 0.075, P < 0.1, P < 0.25, P < 0.5, P < 0.75, and complete set. [file 12870_2022_3479_MOESM2_ESM.tiff]
